# Beyond MPOWER: a systematic review of population-level factors that affect European tobacco smoking rates

**DOI:** 10.1093/eurpub/ckad112

**Published:** 2023-07-27

**Authors:** Leah K Watson, Isaac Weldon, Gigi O Lin, Tina Nanyangwe-Moyo, Steven J Hoffman, Mathieu J P Poirier

**Affiliations:** Global Strategy Lab, Dahdaleh Institute for Global Health Research, Faculty of Health and Osgoode Hall Law School, York University, Toronto, ON, Canada; Global Strategy Lab, Dahdaleh Institute for Global Health Research, Faculty of Health and Osgoode Hall Law School, York University, Toronto, ON, Canada; Department of Politics, York University, Toronto, ON, Canada; Global Strategy Lab, Dahdaleh Institute for Global Health Research, Faculty of Health and Osgoode Hall Law School, York University, Toronto, ON, Canada; Global Strategy Lab, Dahdaleh Institute for Global Health Research, Faculty of Health and Osgoode Hall Law School, York University, Toronto, ON, Canada; Global Strategy Lab, Dahdaleh Institute for Global Health Research, Faculty of Health and Osgoode Hall Law School, York University, Toronto, ON, Canada; School of Global Health, Faculty of Health, York University, Toronto, ON, Canada; Department of Health Research Methods, Evidence, and Impact, McMaster Health Forum, McMaster University, Hamilton, ON, Canada; Global Strategy Lab, Dahdaleh Institute for Global Health Research, Faculty of Health and Osgoode Hall Law School, York University, Toronto, ON, Canada; School of Global Health, Faculty of Health, York University, Toronto, ON, Canada

## Abstract

**Background:**

Population-level factors within and beyond the scope of the World Health Organization’s (WHO) MPOWER policy package have significant impacts on smoking rates. However, no synthesis of the existing evidence exists. This systematic review identifies population-level factors that influence cigarette smoking rates in European countries.

**Methods:**

We searched the ProQuest database collection for original, peer-reviewed quantitative evaluations that investigated the effects of population-level exposures on smoking rates in European countries. Of the 3122 studies screened, 62 were ultimately included in the review. A standardized data extraction form was used to identify key characteristics of each study including publication year, years evaluated, countries studied, population characteristics, study design, data sources, analytic methods, exposure studied, relevant covariates and effects on tobacco smoking outcomes.

**Results:**

One hundred and fifty-five population-level exposures were extracted from the 62 studies included in the review, 99 of which were related to WHO MPOWER measures. An additional 56 exposures fell into eight policy realms: economic crises, education policy, macro-economic factors, non-MPOWER tobacco regulations, population welfare, public policy, sales to minors and unemployment rates. About one-half of the MPOWER exposures affected smoking rates (55/99) and did so in an overwhelmingly positive way (55/55). Over three-quarters of the non-MPOWER exposures were associated with statistically significant changes in smoking outcomes (43/56), with about two-thirds of these exposures leading to a decrease in smoking (29/43).

**Conclusions:**

Population-level factors that fall outside of the WHO’s MPOWER measures are an understudied research area. The impacts of these factors on tobacco control should be considered by policymakers.

## Introduction

The severe health, economic and social impacts of tobacco have generated significant efforts to better understand and address the drivers of its consumption.^1^^,^^2^ European smoking trends in particular have attracted significant research and policy attention because of the region’s persistent investments in tobacco research and historically high smoking rates.^2^^,^^3^ This research has demonstrated that despite sharing similar geographic and, in some cases, political characteristics (e.g. accession to the European Union), countries in the World Health Organization (WHO) European region display varied tobacco consumption trajectories. More specifically, many countries in the European region have been experiencing declines in smoking rates for multiple decades;^4^ however, worrying trends are occurring in some countries and subpopulations, where smoking has stabilized or even increased.^5^ As tobacco consumption continues to be a leading cause of morbidity and mortality in Europe, these concurrent trends underscore the need for further research and interventions to curb the region’s evolving smoking epidemic.^5^^,^^6^

Decades of investments in national statistical agencies and tobacco research have produced more reliable, comparable and publicly available data for Europe than any other WHO region. Some studies have leveraged these large datasets to establish a relationship between European smoking trends and national-level regulations, demonstrating that population-level factors and interventions have a significant role to play in reducing smoking rates and mortality.^7^^,^^8^ International tobacco regulations have also been investigated as policy levers to strengthen national mechanisms for enforcing tobacco policy.^4^^,^^9–11^ The most important example is WHO’s Framework Convention on Tobacco Control (FCTC), which entered into force on 27 February 2005 after more than 5 years of negotiations.^12^ To support the FCTC’s implementation, WHO introduced its ‘MPOWER’ policy package in 2008, comprising six broad measures designed to reduce tobacco demand (Supplementary file S1).^13^

The impact of the FCTC and WHO’s MPOWER measures have been well-described in academic literature. Previous studies and reviews generally support the effectiveness of these measures in reducing smoking prevalence,^9^^,^^14^^,^^15^ cigarette consumption^4^ and smoking-attributable deaths^13^ when implemented at the national level, with especially notable declines observed across Europe.^4^ Beyond the scope of these MPOWER measures, there are numerous population-level factors whose impacts on smoking rates have been studied; however, no studies to our knowledge have systematically investigated and synthesized the evidence of the impacts of these factors.

In this systematic review, we investigate national and multi-country studies to catalogue population-level factors identified in peer-reviewed literature that affect smoking rates in European countries, elucidating both MPOWER-related and non-MPOWER-related factors. In doing so, we make a case for strengthening data availability and robust research across a variety of domains related to tobacco consumption. We further argue that broadening the horizon of possible policy interventions to reduce smoking, beyond those promoted in the WHO’s MPOWER policy package, may help to advance tobacco control efforts.

## Methods

### Search strategy

After consulting with a York University academic librarian, we searched the ProQuest collection of 65 constituent databases, which encompasses a wide range of topics and disciplines including biomedical and public health, sociology, economics and others (Supplementary file S2). Search dates ranged from the date of individual database inception until 20 November 2019. The search was performed without language or date restrictions. All studies were screened using Rayyan systematic review software in two stages. First, two authors (L.K.W. and I.W.) independently screened titles and abstracts in duplicate using the inclusion criteria; second, two authors (L.K.W. and I.W.) independently screened the included full texts in duplicate. Disagreements were resolved via discussion and through consultation with a third rater (M.J.P. or G.O.L.) if necessary.

Studies were included in this systematic review if they: conducted an original quantitative evaluation at the national or cross-national population level; evaluated the effect(s) of one or more population-level exposures on one or more measures of smoking rates; used data from at least one country in the WHO European region; reported findings at the country level; and were peer-reviewed publications.

Studies were excluded from this systematic review if they only described smoking or tobacco consumption prevalence patterns; only reported findings at a level lower than the country level or reported multi-country findings that could not be disaggregated at the country level for European countries; addressed outcomes only related to use of non-tobacco or electronic cigarette products; only looked at individual- or community-level exposures; or were exclusively modelling studies.

### Data extraction

A standardized data extraction form was developed and applied for this review (Supplementary file S3). Two extractors (L.K.W. and I.W.) completed the data extraction form related to the key characteristics of each study, including: publication year, years in which the study was conducted, European countries studied, population characteristics, study design, data sources, analytic methods, exposures and outcomes of interest, relevant covariates and whether the study authors identified a significant effect of the exposure on the outcome.

Smoking-related outcomes were grouped into three categories: smoking prevalence, which represents the number of individuals who smoke in a population; smoking intensity, which represents how often individuals who smoke participate in smoking; and population-level cigarette consumption, an aggregate indicator of regional or national purchases of tobacco products. Outcomes were also categorized as investigating only youth (under 18 years old), only adults (18 years and older), or the general population (no specified age range, or encompassing both youth and adults).

### Assessment of study quality

Two authors (L.K.W. and I.W.) assessed study quality using the Joanna Briggs Institute (JBI) critical appraisal materials for the appropriate study design.^16^ Since no JBI checklist exists for time-series studies, the authors modified an existing JBI checklist by adding three additional questions based on the Cochrane EPOC risk of bias tool for time-series studies.^17^ Finally, we repeated analyses after removing studies that did not meet at least 50% of the criteria outlined in their relevant quality assessment tool to evaluate the robustness of our findings.

## Results

**Figure 1:**
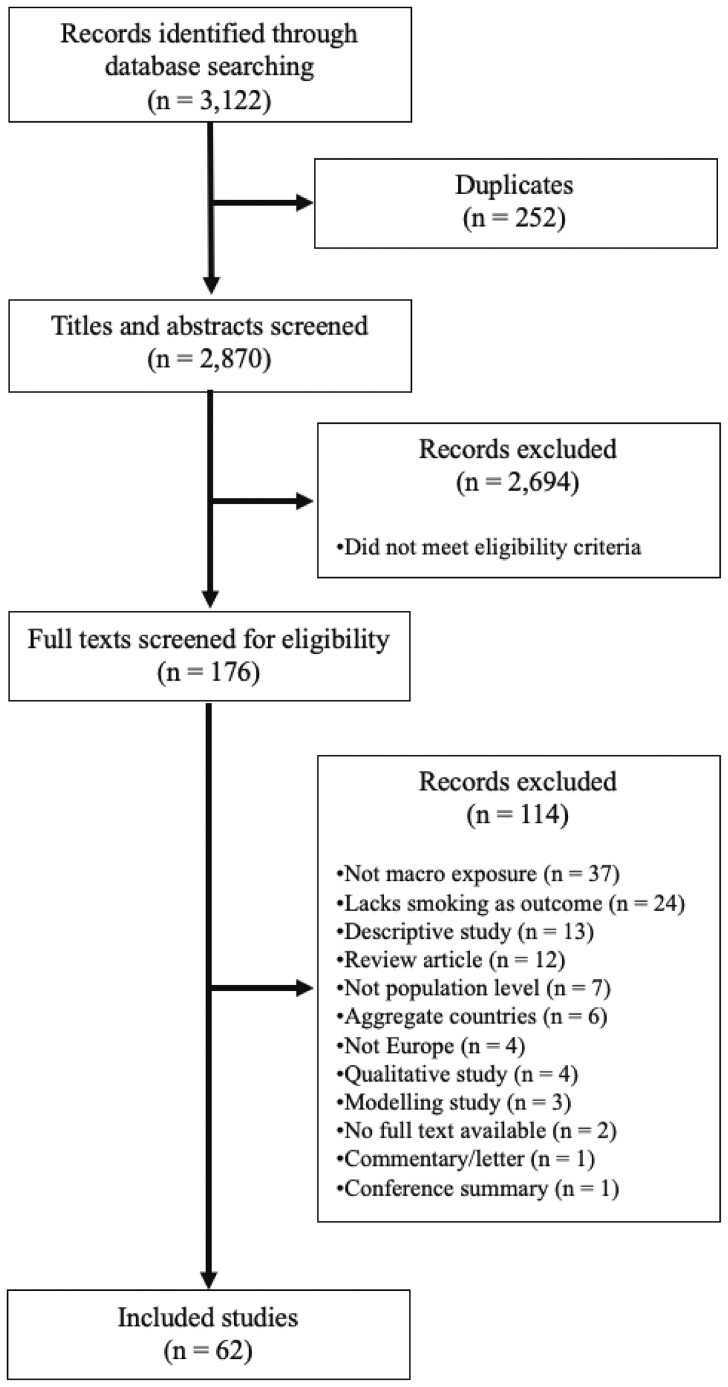
PRISMA flow chart

Our initial search yielded 3122 results. After removing duplicates, 2870 titles and abstracts were screened and 176 articles were selected for full review (figure 1). Full texts of these 176 studies were independently screened in duplicate, and 62 studies were ultimately included (Supplementary file S4). The included studies were generally of high quality, with an average risk of bias score of 78% (Supplementary file S5). A total of 155 population-level exposures related to smoking rates were extracted from these studies. These exposures were categorized into a relevant MPOWER category if applicable, regardless of whether the study’s authors classified them as such or if their data were sourced from official reports to the WHO FCTC Secretariat (Supplementary file S1). Any exposures that did not align with an MPOWER measure were sorted into one of eight non-MPOWER policy realms (Supplementary file S6).

In the overall dataset, ∼60% (99/155) of the exposures were found to be significantly associated with a change in smoking rates. A summary of included studies and key extracted variables can be found in Supplementary file S7.

### Non-MPOWER exposures

**Figure 2:**
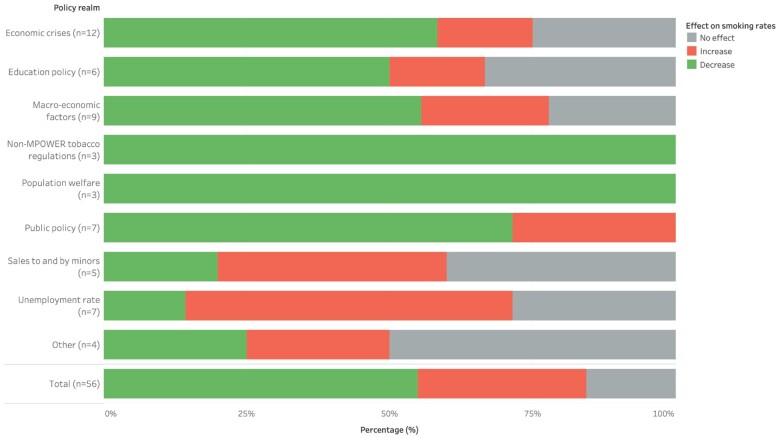
Impacts of non-MPOWER-related exposures on smoking rates, by policy realm

Of the 155 exposures identified in this review, 56 exposures fell outside the scope of the FCTC’s MPOWER measures and were categorized into the policy realms of economic crises, education policy, macro-economic factors, non-MPOWER tobacco regulations, population welfare, public policy, sales to and by minors and unemployment rate (Supplementary file S8). Four of the 56 non-MPOWER exposures that did not fall within these realms were categorized as ‘other’. Approximately three-quarters of the non-MPOWER exposures (43/56) were statistically significantly associated with smoking rates. The majority of these were linked to a decrease (29/56), 14/56 were associated with an increase and the remaining 13/56 were not significantly associated with smoking rates (figure 2).

The most common non-MPOWER policy realms in the dataset were economic crises and macro-economic indicators, which together comprised 21 of the 56 non-MPOWER exposures. Economic crises, such as the 2008 recession in Iceland, the Netherlands, Greece and Italy, were largely found to decrease tobacco consumption (7/12). The remaining two studies associated with increases and three not associated with significant changes all evaluated Iceland’s 2008 economic recession. Similarly, improvements in macro-economic indicators, including higher economic output (e.g. GDP and GNI) and lower economic inequality (e.g. Gini coefficient), were mostly (5/9) found to decrease smoking rates, but 2/9 were associated with increases and another 2/9 were not found to be statistically significant. Meanwhile, four of the seven exposures related to increases in national, regional, or youth-specific unemployment rates were found to increase smoking rates, while one was associated with decreased smoking and two found no association.

Public policy is a more diverse category, spanning increased government spending on social and health programmes and low perception of corruption. Despite its heterogeneity, 5/7 exposures in this category were associated with decreased smoking rates, with only two evaluations of the German Kindergeld parental benefit finding associations with increased smoking. Similarly, three of the six exposures related to national education policy and population-level educational achievement were linked to decreases in smoking rates. Only one study of post-war education reforms found that it increased smoking rates and two others found no association. Broader population welfare measures of life satisfaction, gender equality and human development were all associated with decreased tobacco consumption.

Exposures related to tobacco sales to minors, including minimum purchasing age and vending machine policies, were unexpectedly linked to smoking rate increases in two of five studies, with only one study finding a decrease in rates, and two finding no significant impacts. These exposures are related to FCTC Article 16 on the supply and exposure of tobacco to minors but are not specifically included in WHO’s six MPOWER measures.^18^ Unspecified tobacco regulations, such as the presence of tobacco regulations or laws, were linked to a decrease in smoking rates in all three studies in this category. Finally, residence in the former Eastern Bloc was associated with increased smoking, residence in Western Europe was associated decreased smoking and national tobacco production and population-level religiosity were not associated with tobacco consumption.

### MPOWER exposures

**Figure 3:**
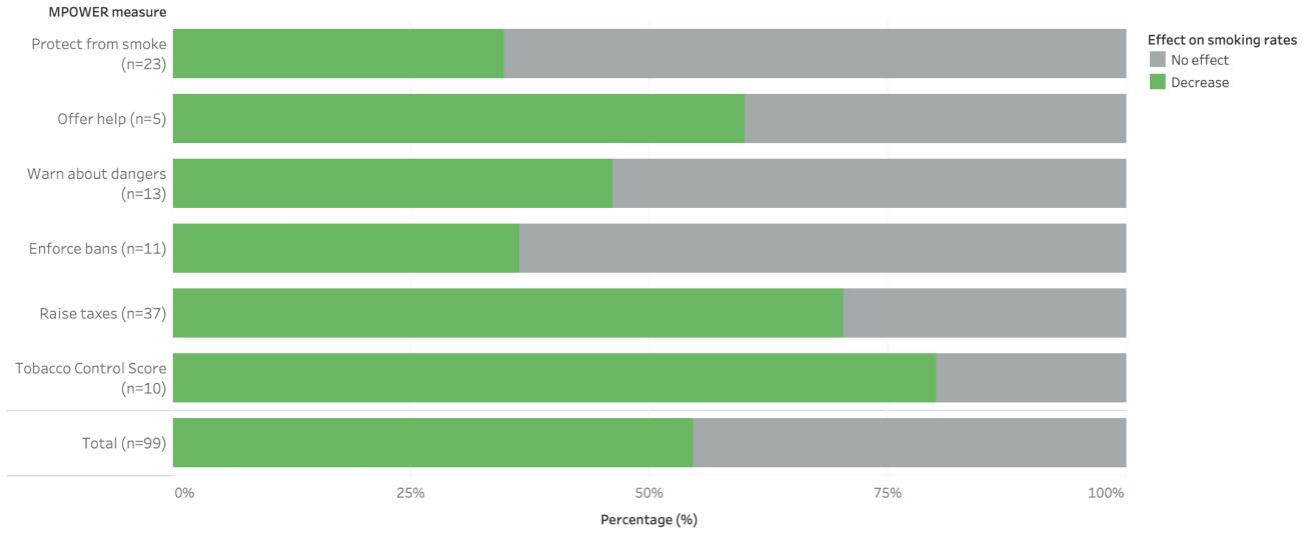
Impacts of MPOWER exposures on smoking rates, by MPOWER measure

Nearly two-thirds of the exposures (99/155) were related to the FCTC’s MPOWER measures, of which 10 outcomes were measured only in subpopulations (Supplementary file S9). Over half of these measures were found to influence smoking (55/99), with every single significant effect associated with decreased smoking rates (55/55) (figure 3). Five of the six MPOWER measures were reflected by at least one study exposure. The only MPOWER measure that was not directly assessed for population-level impact was ‘M’—monitoring tobacco use and prevention policies—which aims to strengthen tobacco-related data quality and data collection capacity.

‘R’—raising cigarette prices and increasing taxation—was the most studied MPOWER measure, for which 26 out of the 37 findings were significantly associated with decreased smoking rates. The next most common measure was ‘P’—protection from tobacco smoke through the establishment of smoke-free workplaces, schools and public spaces. This measure was far less likely to be associated with changes in smoking rates (8/23). ‘W’—warning about the dangers of tobacco using public health education and messaging—was associated with reductions in smoking in 6 of 13 outcomes. Meanwhile, only 4 of the 11 exposures related to ‘E’—enforcing bans on tobacco advertising, promotion and sponsorship—were linked to a decrease in smoking rates. The least frequently investigated MPOWER measure was ‘O’—offering programmatic and financial support for individuals who attempt to quit smoking—with three of the five exposures associated with significant decreases in smoking. Lastly, a country-level Tobacco Control Score compiling actions on all six MPOWER measures was consistently associated with decreased tobacco consumption, with 8 of the 10 outcomes finding significant reductions in smoking.

### Outcomes, countries and populations studied

The most common outcome studied was smoking prevalence (112/155), followed by population-level cigarette sales (27/155) and smoking intensity (14/155). Two outcomes did not fall into these categories: smoking-related behavioural response aggregating both smoking prevalence and intensity, and smoking behaviours at home. Both smoking prevalence and cigarette sales were found to be more consistently associated with population-level exposures than smoking intensity, with 71/112 significant associations for smoking prevalence, 19/27 significant associations for cigarette sales and 7 of 14 significant associations for smoking intensity.

**Figure 4:**
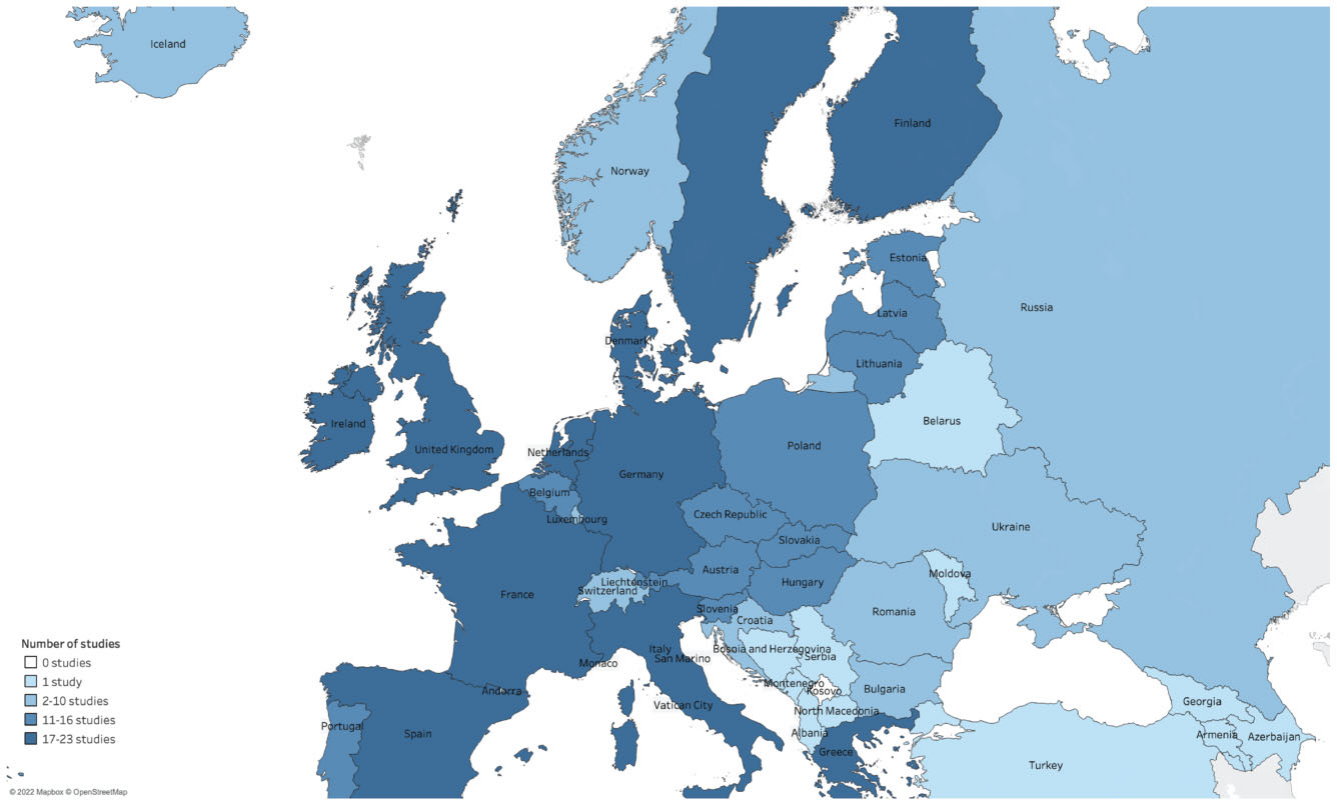
Distribution of European countries in included studies

The 62 included studies covered nearly all countries in Europe, with Western European countries generally overrepresented in the dataset (figure 4). The UK was the most frequently studied country (*n* = 23), followed by Germany, Ireland and Spain (*n* = 20); France and Italy (*n* = 19); and Greece, the Netherlands and Sweden (*n* = 18).

Just over half of the included findings (83/155) evaluated changes among the general population, while 48/155 focused on adults only and 24/155 evaluated youth only. Outcomes measured among adults only were just as likely to be influenced by population-level exposures (32/48) as those measured among the general population (53/83) but were more likely to be associated with increases in smoking (Supplementary file S10). In contrast, outcomes measured in youth-only populations were less likely to be influenced by population-level exposures (13/24) but less likely to increase smoking than the adult age group. Finally, MPOWER, non-MPOWER and age category results were not changed after removing studies that failed to meet at least 50% of the criteria outlined in their relevant quality assessment tool (Supplementary file S11).

## Discussion

### Principal findings

This study contextualizes the effectiveness of MPOWER measures relative to other, less frequently studied factors that impact population-level smoking rates. Although MPOWER exposures were less likely to influence smoking rates compared to non-MPOWER factors, when they did have an impact, it was overwhelmingly positive. Our findings align with the preponderance of evidence supporting the effectiveness of raising taxes on tobacco as being the most consistently effective MPOWER measure.^19^^,^^20^ Exposures that fell outside the scope of the WHO’s MPOWER package varied greatly in their impact, with approximately half of exposures leading to a decrease in tobacco consumption, a quarter leading to increases and a quarter lacking significant association. Our findings were consistent across multiple study types, data sources and robustness checks, reducing the potential for confirmation bias. These results imply that, alongside MPOWER measures, the most important policy priorities for reducing population-level smoking rates should include improving population welfare, public policy and macro-economic indicators, as well as reducing unemployment rates.

Although our findings highlight the importance of population-level factors as determinants of smoking rates, there are relatively few attempts to synthesize evidence of these effects and make policy recommendations to address these factors. In addition to the relatively well-studied MPOWER measures, the eight policy realms and 56 population-level exposures that were found to impact smoking rates can be prioritized in future research (Supplementary file S8). For example, researchers could investigate the impacts of social programmes, educational reform, or employment insurance on tobacco consumption even if it is not the primary objective of the policy.

The geographical distribution of the studies and, relatedly, of the datasets used to answer research questions indicate a research bias towards Western European countries as compared to Eastern European countries. Surveys, such as the Eurobarometer and Health Behaviour in School Aged Children, appeared frequently in included studies, but many studies used data sources limited to one or a few countries, including national-level taxation and sales records or public health surveys conducted at the national level. These findings support claims of unbalanced smoking prevalence measurement and analysis across Europe.^21^ Overall, there is a need for increased research on the effect of population-level exposures on smoking rates in Eastern European countries, ideally using internationally standardized datasets to facilitate comparative analyses with other European and global regions.

Finally, our dataset consisted predominantly of studies that analyzed findings for either the general population or for adults aged 18 years and older. Only about 16% of exposures were evaluated for impacts on youth populations, and these were less likely to lead to decreases than among adults or the general population. Youth smoking rates in Europe tend to be high compared to the rest of the world and have displayed some worrying increasing trends in recent years.^6^^,^^22^ It is therefore crucial to continue research efforts exploring youth smoking to better understand the effects of population-level factors, policies and socio-economic determinants on these trends.

### Strengths and limitations

To our knowledge, this is the first systematic review to compile a comprehensive set of population-level factors linked to national tobacco consumption. Consequently, this is the first synthesis of evidence of the impacts of population-level exposures that are not included in WHO’s MPOWER package on tobacco consumption. Finally, the review drew on research from 65 multidisciplinary databases to elucidate factors that affect tobacco consumption in European countries. Each study was reviewed in duplicate, data were extracted using a standardized form, and quality for each included study was assessed using JBI checklists.

One weakness is that our search yielded some studies over 15 years old with poorly described study designs, which limited our ability to analyze their statistical methods. While the included studies were generally high quality, many cross-sectional and some time-series studies either did not analyze or report their methods for addressing confounding variables. This may affect inferences made by some studies about the true relationship between the exposures of interest and smoking rates. Over half of the studies included in this review (*n* = 35) used cross-sectional or ecological designs, suggesting that this area of research would benefit from studies with longer time horizons that could be used to establish causal relationships between smoking outcomes and population-level exposures of interest. Policy realms, such as public policy measures and measures of population welfare, may especially benefit from longer-term studies, as the measurement of these exposures frequently requires longer timeframes to elucidate their impact on smoking rates. Another weakness is that while the MPOWER measure of protecting people from tobacco smoke appears to be the least likely to affect smoking rates, this measure is more appropriately assessed in relation to the harms of second-hand smoke, which was beyond the ambit of our search.

### Policy implications

This systematic review of population-level factors that affect smoking in European countries suggests three ways to strengthen European tobacco policy. First, policy realms that fall outside WHO’s MPOWER measures can form an important part of national tobacco control strategies and further strengthen the FCTC’s health in all policies approach to tobacco control.^23^ Improving social and economic welfare through public policy may be as important as raising the price of tobacco and offering help to quit smoking. Second, assessing policy interventions depends upon the regular collection and availability of population-level data to enable evidence-informed policy measures for maximum effectiveness. Countries should continue supporting tobacco research, publishing data on determinants of smoking and standardizing metrics to facilitate cross-country comparison. Third, while our findings support the continued use of MPOWER measures, they also indicate that some MPOWER measures, such as price and tax increases may be more effective than others.

Governments could expand the breadth of their tobacco control measures by, e.g. improving social protection schemes, reducing economic inequality and improving overall access to and quality of education. Relatedly, the potential for non-tobacco-related public and social policy to lower tobacco consumption means that the true social and economic value of these interventions is most likely underestimated due to positive externalities. In other words, public and social policy interventions that also reduce smoking rates, such as educational reform, provide a social benefit by reducing the costs associated with tobacco-related illness. If these benefits can be quantified, then they should also be accounted for when conducting cost-benefit analyses of new social programmes to inform public policy.

### Directions for future research

Tobacco control research continues to play a critical role in helping governments craft policies that mitigate the negative health, economic and social impacts of smoking. Our findings highlight a significant imbalance in the geographic distribution of studies, suggesting that future research should focus on building a stronger evidence base for Eastern Europe. Additionally, non-MPOWER measures represent a set of promising yet under investigated strategies to reduce smoking rates, and our findings suggest that there are several policy areas deserving further investigation. Finally, given that many policy interventions yielded mixed effects, there is a critical need for studies that investigate the conditions under which both MPOWER and non-MPOWER measures are most likely to work and achieve their maximal effectiveness.

## Supplementary Material

ckad112_Supplementary_Data

## Data Availability

The authors confirm that the data supporting the findings of this study are available within the article and its Supplementary materials. This is the first synthesis of the existing evidence on the impacts of population-level factors that influence smoking rates in Europe, including those that fall within WHO’s MPOWER package and those that fall outside its scope. Measures that fall outside the WHO’s MPOWER policy package can form an important part of national tobacco control strategies and further strengthen the WHO and its Framework Convention on Tobacco Control’s health in all policies approach to tobacco control. Research on tobacco control should continue to investigate the conditions that maximize the effectiveness of MPOWER measures and other population factors for reducing smoking rates.
