# Supplementary material for: Beyond MPOWER: a systematic review of population-level factors that affect European tobacco smoking rates
Source: Eur J Public Health. 2023 Jul 27;33(5):851–6. doi: 10.1093/eurpub/ckad112 (PMC10567248; doi:10.1093/eurpub/ckad112)
Supplement: ckad112_Supplementary_Data [file ckad112_supplementary_data.pdf]

Supplementary File 1: MPOWER measures and descriptions

MPOWER measure	Description
1. **M**onitoring tobacco use and prevention policies	Strengthening the measurement and assessment capacity of countries to improve data quality and coverage and to reinforce policy implementation.
2. **P**rotect people from tobacco smoke	Encouraging implementation of smoke-free laws in public places and places of work.
3. **O**ffer help to quit tobacco use	Establishing and funding programs to assist individuals making quit attempts.
4. **W**arn about the dangers of tobacco	Implementing and bolstering public health education and targeted messaging, e.g., warnings on tobacco packaging.
5. **E**nforce bans on tobacco advertising, promotion, and sponsorship	Reducing the public's exposure to the influence of tobacco companies by preventing public pro-tobacco messaging.
6. **R**aise taxes on tobacco	Encouraging national implementation of tobacco taxation to reduce individual-level purchasing and to boost funding for tobacco control initiatives.

Measures from: World Health Organization. WHO Report on the Global Tobacco Epidemic, 2008: the MPOWER package. Geneva, Switzerland: World Health Organization 2008. https://apps.who.int/iris/handle/10665/43818 (accessed 27 Oct 2021).

Supplementary File 2: Keywords and ProQuest databases included in systematic review search (n = 65)

Keywords:

(“Europe*”) AND (“Macro*” OR “Global” OR “Socio-economic” OR “Social” OR “Societal” OR “Econom*” OR “Education*” OR “Population*” OR “Cultur*” OR “Religi*”) AND (“Smoking” OR “Cigarette consumption” OR “Tobacco Use”)

Databases:

	Title	Subject Area in ProQuest
1.	ABI/INFORM Global	Business
2.	ABI/INFORM Trade & Industry	Business
3.	American Periodicals	Literature & Language, Social Sciences, History, The Arts
4.	The Annual Register: A Record of World Events	Literature & Language, Social Sciences, History
5.	APA PsychArticles	Social Sciences, Health & Medicine
6.	APA PsycBooks	Social Sciences, Health & Medicine
7.	APA PsycInfo	Social Sciences, Health & Medicine
8.	Applied Social Sciences Index & Abstracts (ASSIA)	Social Sciences
9.	ARTbibliographies Modern (ABM)	The Arts
10.	Asian & European Business Collection	(unassigned)
11.	Australian Education Index	(unassigned)
12.	Avery Index to Architectural Periodicals	The Arts
13.	Black Abolitionist Papers	Social Sciences, History
14.	Black Short Fiction and Folklore	Literature & Language, The Arts
15.	Black Studies Center	Social Sciences, History
16.	Black Women Writers	Literature & Language, The Arts
17.	British Periodicals	Literature & Language ,Social Sciences, History, The Arts
18.	Canadian Business & Current Affairs Database	(unassigned)
19.	Canadian Major Dailies	News & Newspapers
20.	Canadian Research Index	Social Sciences
21.	Caribbean Literature	Literature & Language, The Arts
22.	Colonial State Papers	History
23.	Design & Applied Arts Index (DAAI)	The Arts
24.	Dissertations & Theses @ York University	Dissertations & Theses
25.	Documents on British Policy Overseas	History
26.	Early Modern Books	Literature & Language

	Early English Books Online	*Literature & Language*
	Early European Books	*Literature & Language*
27.	Ebook Central	(unassigned)
28.	EconLit	Social Sciences
29.	Entertainment Industry Magazine Archive	History, The Arts
30.	ERIC	Social Sciences
31.	Ethnic NewsWatch	News & Newspapers, Literature & Language, Social Sciences, History, The Arts
32.	Humanities Index	Literature & Language, History, The Arts
33.	Index Islamicus	Literature & Language, Social Sciences, History, The Arts
34.	International Bibliography of Art (IBA)	The Arts
35.	International Bibliography of the Social Sciences (IBSS)	Social Sciences
36.	Latin American Women Writers	Literature & Language, The Arts
37.	Latino Literature: Poetry, Drama, and Fiction	Literature & Language, The Arts
38.	LGBT Magazine Archive	Social Sciences, History
39.	Library & Information Science Abstracts (LISA)	Science & Technology, Social Sciences
40.	Linguistics and Language Behavior Abstracts (LLBA)	Literature & Language, Social Sciences
41.	Literature Online	Literature & Language
42.	Music & Performing Arts Collection	The Arts
	Music Periodicals Database	*The Arts*
	Performing Arts Periodicals Database	*Literature & Language, The Arts*
43.	Nursing & Allied Health Premium	Health & Medicine
44.	Periodicals Archive Online	Literature & Language, Social Sciences, History, The Arts
45.	Philosopher's Index	Social Sciences, History, The Arts
46.	Politics Collection	Social Sciences
	PAIS Index	*Social Sciences*
	Policy File Index	*Social Sciences*
	Political Science Database	*Social Sciences*
	Worldwide Political Science Abstracts	*Social Sciences*
47.	PRISMA Database with HAPI Index	Literature & Language, Social Sciences, History, The Arts
48.	ProQuest Dissertations & Theses Global	Dissertations & Theses
49.	ProQuest Historical Newspapers: Atlanta Daily World	News & Newspapers, History
50.	ProQuest Historical Newspapers: Chicago Defender	News & Newspapers, History
51.	ProQuest Historical Newspapers: The Globe and Mail	News & Newspapers, History

52.	ProQuest Historical Newspapers: Los Angeles Sentinel	News & Newspapers, History
53.	ProQuest Historical Newspapers: The New York Times	News & Newspapers, History
54.	ProQuest Historical Newspapers: The Times of India	News & Newspapers, History
55.	ProQuest Historical Newspapers: Toronto Star	News & Newspapers, History
56.	PTSDpubs	Social Sciences, Health & Medicine
57.	Public Health Database	Health & Medicine
58.	Publicly Available Content Database	(unassigned)
59.	Screen Studies Collection	Literature & Language, History, The Arts
	AFI Catalog	*Literature & Language, History, The Arts*
	FIAF International Index to Film Periodicals Database	*Literature & Language, History, The Arts*
	Film Index International	*Literature & Language, History, The Arts*
60.	Sociology Collection	Social Sciences
	Applied Social Sciences Index & Abstracts (ASSIA)	*Social Sciences*
	Sociological Abstracts	*Social Sciences*
	Sociology Database	*Social Sciences*
61.	South and Southeast Asian Literature in English	Literature & Language, The Arts
62.	Sports Medicine & Education Index	Science & Technology, Social Sciences, Health & Medicine
63.	Teatro Español del Siglo de Oro	Literature & Language
64.	Twentieth-Century Drama	Literature & Language
65.	Women’s Magazine Archive	Social Sciences, History, The Arts

Supplementary File 3: Variables extracted for systematic review

First author	
Publication year	
Study period (years)	
Country/countries (European region only)	
Study population (broad)	*General population / Adults only (18+ years) / Youth only (<18 years)*
Study population (age range)	
Sample size	
Study design	
Dataset(s) used	
Analytic method(s)	
Independent variable/exposure	
	MPOWER / Non-MPOWER
Covariates	
Dependent variable (broad)	*Smoking prevalence / Smoking intensity / Cigarette sales / Other*
Dependent variable (specific)	
Author-determined presence of effect	*Yes / Sometimes / No*
	If Yes/Sometimes: *Increase in smoking rates / Decrease in smoking rates*
Quantitative effect estimate	
Results (short summary)	

Supplementary File 4: Citations for included studies (n = 62)

Agaku IT, Filippidis FT, Vardavas CI. Effectiveness of Text versus Pictorial Health Warning Labels and Predictors of Support for Plain Packaging of Tobacco Products within the European Union. Eur Addict Res 2015;21:47–52. Doi:10.1159/000366019

Alpert HR, Vardavas CI, Chaloupka FJ, et al. The recent and projected public health and economic benefits of cigarette taxation in Greece. Tob Control 2014;23:452–4. Doi:10.1136/tobaccocontrol-2012-050857

Ásgeirsdóttir TL, Corman H, Noonan K, et al. Was the economic crisis of 2008 good for Icelanders? Impact on health behaviors. Economics & Human Biology 2014;13:1–19. Doi:10.1016/j.ehb.2013.03.005

Ásgeirsdóttir TL, Corman H, Noonan K, et al. Lifecycle effects of a recession on health behaviors: Boom, bust, and recovery in Iceland. Economics & Human Biology 2016;20:90–107. Doi:10.1016/j.ehb.2015.11.001

Basu A, Jones AM, Dias PR. Heterogeneity in the impact of type of schooling on adult health and lifestyle. Journal of Health Economics 2018;57:1–14. Doi:10.1016/j.jhealeco.2017.10.007

Bogdanovica I, McNeill A, Murray R, et al. What Factors Influence Smoking Prevalence and Smoke Free Policy Enactment across the European Union Member States. PloS ONE 2011;6:e23889. Doi:10.1371/journal.pone.0023889

Bogdanovica I, Murray R, McNeill A, et al. Cigarette price, affordability and smoking prevalence in the European Union: Affordability and prevalence. Addiction 2012;107:188–96. Doi:10.1111/j.1360-0443.2011.03588.x

Brüderl, J, Ludwig V. Does a Smoking Ban Reduce Smoking? Evidence from Germany. Schmollers Jahrbuch 2011;131:419–29. Doi:10.3790/schm.131.2.419

Bruggink J-W, de Goeij MCM, Otten F, et al. Changes between pre-crisis and crisis period in socioeconomic inequalities in health and stimulant use in Netherlands. Eur J Public Health 2016;26:772–7. Doi:10.1093/eurpub/ckw016

Chyderiotis S, Beck F, Andler R, et al. How to reduce biases coming from a before and after design: the impact of the 2007–08 French smoking ban policy. European Journal of Public Health 2019;29:372–7. Doi:10.1093/eurpub/cky160

Ciccarelli C. The Consumption of Tobacco in Italy: National and Regional Estimates, 1871-1913. Rivista di storia economica 2012;3 409-452.

Costa J, Mossialos E. Which policies effectively enable smoking cessation? Evidence from the European Union. Int J of Social Economics 2006;33:77–100. Doi:10.1108/03068290610636442

Del Bono E, Vuri D. Smoking behaviour and individual well-being: a fresh look at the effects of the 2005 public smoking ban in Italy. Oxford Economic Papers 2018;70:741–62. Doi:10.1093/oep/gpx039

Di Pietro G. Revisiting the impact of macroeconomic conditions on health behaviours. Economics & Human Biology 2018;28:173–81. Doi:10.1016/j.ehb.2017.11.001

Duffy M. Tobacco consumption and policy in the United Kingdom. Applied Economics 2006;38:1235–57. Doi:10.1080/00036840500392599

Escario JJ, Molina * JA. Will a special tax on tobacco reduce lung cancer mortality? Evidence for EU countries. Applied Economics 2004;36:1717–22. Doi:10.1080/0003684042000266883

Etilé F, Jones AM. Schooling and smoking among the baby boomers – An evaluation of the impact of educational expansion in France. Journal of Health Economics 2011;30:811–31. Doi:10.1016/j.jhealeco.2011.05.002

Feliu A, Filippidis FT, Joossens L, et al. Impact of tobacco control policies on smoking prevalence and quit ratios in 27 European Union countries from 2006 to 2014. Tob Control 2018;:tobaccocontrol-2017-054119. Doi:10.1136/tobaccocontrol-2017-054119

Filippidis FT, Schoretsaniti S, Dimitrakaki C, et al. Trends in cardiovascular risk factors in Greece before and during the financial crisis: the impact of social disparities. The European Journal of Public Health 2014;24:974–9. Doi:10.1093/eurpub/cku028

Florkowski WJ, McNamara KT. Policy implications of alcohol and tobacco demand in Poland. Journal of Policy Modeling 1992;14:93–8. Doi:10.1016/0161-8938(92)90025-8

Forster M, Jones AM. The role of tobacco taxes in starting and quitting smoking: Duration analysis of British data. Journal of the Royal Statistical Society: Series A (Statistics in Society) 2001;164:517–47. Doi:10.1111/1467-985X.00217

Gallus S. Price and cigarette consumption in Europe. Tobacco Control 2006;15:114–9. Doi:10.1136/tc.2005.012468

Hallingberg B, Fletcher A, Murphy S, et al. Do stronger school smoking policies make a difference? Analysis of the health behaviour in school-aged children survey. Eur J Public Health 2016;26:964–8. Doi:10.1093/eurpub/ckw093

Hanewinkel R, Radden C, Rosenkranz T. Price increase causes fewer sales of factory-made cigarettes and higher sales of cheaper loose tobacco in Germany. Health Econ 2008;17:683–93. Doi:10.1002/hec.1282

Hublet A, Schmid H, Clays E, et al. Association between tobacco control policies and smoking behaviour among adolescents in 29 European countries. Addiction 2009;104:1918–26. Doi:10.1111/j.1360-0443.2009.02686.x

Hyland A, Hassan LM, Higbee C, et al. The impact of smokefree legislation in Scotland: results from the Scottish ITC Scotland/UK longitudinal surveys. The European Journal of Public Health 2009;19:198–205. Doi:10.1093/eurpub/ckn141

James J. Health and education expansion. Economics of Education Review 2015;49:193–215. Doi:10.1016/j.econedurev.2015.10.003

Jiménez-Martín S, Labeaga JM, López A. Participation, heterogeneity and dynamics in tobacco consumption: evidence from cohort data. Health Econ 1998;7:401–14. Doi:10.1002/(sici)1099-1050(199808)7:5<401::aid-hec361>3.0.co;2-2

Jones AM, Laporte A, Rice N, et al. Do Public Smoking Bans have an Impact on Active Smoking? Evidence from the UK: Do Public Smoking Bans have an Impact on Active Smoking? Health Econ 2015;24:175–92. Doi:10.1002/hec.3009

Juerges H, Reinhold S, Salm M. Does Schooling Affect Health Behavior? Evidence from Educational Expansion in Western Germany. SSRN Journal Published Online First: 2009. Doi:10.2139/ssrn.1466935

Kaiser M, Reutter M, Sousa-Poza A, et al. Smoking and local unemployment: Evidence from Germany. Economics & Human Biology 2018;29:138–47. Doi:10.1016/j.ehb.2018.02.004

Kuipers MAG, Brandhof SD, Monshouwer K, et al. Impact of laws restricting the sale of tobacco to minors on adolescent smoking and perceived obtainability of cigarettes: an intervention-control pre-post study of 19 European Union countries: Youth tobacco access laws in Europe. Addiction 2017;112:320–9. Doi:10.1111/add.13605

Lance PM, Akin JS, Dow WH, et al. Is cigarette smoking in poorer nations highly sensitive to price? Journal of Health Economics 2004;23:173–89. Doi:10.1016/j.jhealeco.2003.09.004

Lemennicier B, Maillard O, Scano E. L'impact de la publicité sur la demande. Une application à la demande de tabac en France (1970-1994). Revue économique 1998;49:1539–71. Doi:10.2307/3502623

León-Gómez BB, Colell E, Villalbí JR, et al. Impact of smoke-free regulations on smoking prevalence trends in Spain. Eur J Public Health 2016;:ckw151. Doi:10.1093/eurpub/ckw151

Levy DT, Currie L, Clancy L. Tobacco control policy in the UK: blueprint for the rest of Europe? The European Journal of Public Health 2013;23:201–6. Doi:10.1093/eurpub/cks090

Levy DT, Huang A-T, Currie LM, et al. The benefits from complying with the framework convention on tobacco control: a SimSmoke analysis of 15 European nations. Health Policy and Planning 2014;29:1031–42. Doi:10.1093/heapol/czt085

Lidón-Moyano C, Martín-Sánchez JC, Saliba P, et al. Correlation between tobacco control

policies, consumption of rolled tobacco and e-cigarettes, and intention to quit conventional tobacco, in Europe. Tob Control 2017;26:149–52. Doi:10.1136/tobaccocontrol-2015-052482

Ma Y, Nolan A, Smith JP. The value of education to health: Evidence from Ireland. Economics & Human Biology 2018;31:14–25. Doi:10.1016/j.ehb.2018.07.006

Marti J. The impact of tobacco control expenditures on smoking initiation and cessation: Tobacco control expenditures and smking decisions. Health Econ 2014;23:1397–410. Doi:10.1002/hec.2993

Martin Bassols N, Vall Castelló J. Effects of the great recession on drugs consumption in Spain. Economics & Human Biology 2016;22:103–16. Doi:10.1016/j.ehb.2016.03.005

Nagelhout GE, de Vries H, Boudreau C, et al. Comparative impact of smoke-free legislation on smoking cessation in three European countries. The European Journal of Public Health 2012;22:4–9. Doi:10.1093/eurpub/ckr203

Nicolás AL. How important are tobacco prices in the propensity to start and quit smoking? An analysis of smoking histories from the Spanish National Health Survey: Influence of Tobacco Prices on Smoking. Health Econ 2002;11:521–35. Doi:10.1002/hec.745

Nociar A, Sierosławski J, Csémy L. Substance Use among European Students: East – West Comparison between 1995 and 2011. Cent Eur J Public Health 2016;24:281–8. Doi:10.21101/cejph.a4309

Ogloblin C, Brock G. The Rise in Female Smoking in Russia: What to Do? Regional and Sectoral Economic Studies 2011;11.https://ideas.repec.org/a/eaa/eerese/v11y2011i2_4.html (accessed 11 Jan 2021).

Ogloblin C, Brock G. Smoking in Russia: The ‘Marlboro Man’ Rides but Without ‘Virginia Slims’ for Now. Comp Econ Stud 2003;45:87–103. Doi:10.1057/palgrave.ces.8100001

Ólafsdóttir T, Hrafnkelsson B, Ásgeirsdóttir TL. The Icelandic economic collapse, smoking, and the role of labor-market changes. Eur J Health Econ 2015;16:391–405. Doi:10.1007/s10198-014-0580-x

Palali A, van Ours JC. The impact of tobacco control policies on smoking initiation in eleven European countries. Eur J Health Econ 2019;20:1287–301. Doi:10.1007/s10198-019-01090-x

Peng L, Ross H. The Impact of Cigarette Taxes and Advertising on the Demand for Cigarettes in Ukraine. Cent Eur J Public Health 2009;17:93–8. Doi:10.21101/cejph.a3518

Pförtner T-K, Hublet A, Schnohr CW, et al. Socioeconomic inequalities in the impact of tobacco control policies on adolescent smoking. A multilevel study in 29 European countries. Addictive Behaviors 2016;53:58–66. Doi:10.1016/j.addbeh.2015.09.016

Radfar M. The effect of advertising on total consumption of cigarettes in the U.K. European Economic Review 1985;29:225–31. Doi:10.1016/0014-2921(85)90053-4

Raschke C. The Impact of the German Child Benefit on Household Expenditures and Consumption. German Economic Review 2016;17:438–77. Doi:10.1111/geer.12079

Rathmann K, Pförtner T-K, Elgar FJ, et al. The Great Recession, Adolescent Smoking, and Smoking Inequalities: What Role Does Youth Unemployment Play in 24 European Countries? NICTOB 2016;:ntw298. Doi:10.1093/ntr/ntw298

Ross H, Kostova D, Stoklosa M, et al. The Impact of Cigarette Excise Taxes on Smoking Cessation Rates From 1994 to 2010 in Poland, Russia, and Ukraine. NICTOB 2014;16:S37–43. Doi:10.1093/ntr/ntt024

Savage M. Smoking outside: the effect of the Irish workplace smoking ban on smoking prevalence among the employed. HEPL 2014;9:407–24. Doi:10.1017/S1744133114000036

Schaap MM, Kunst AE, Leinsalu M, et al. Effect of nationwide tobacco control policies on smoking cessation in high and low educated groups in 18 European countries. Tobacco Control 2008;17:248–55. Doi:10.1136/tc.2007.024265

Schnohr CW, Kreiner S, Rasmussen M, et al. The role of national policies intended to regulate adolescent smoking in explaining the prevalence of daily smoking: a study of adolescents from 27 European countries. Addiction 2008;103:824–31. Doi:10.1111/j.1360-0443.2008.02161.x

Serrano-Alarcón M, Kunst AE, Bosdriesz JR, et al. Tobacco control policies and smoking among older adults: a longitudinal analysis of 10 European countries. Addiction 2019;114:1076–85. Doi:10.1111/add.14577

Stavrinos VG. The effects of an anti-smoking campaign on cigarette consumption: empirical evidence from Greece. Applied Economics 1987;19:323–9. Doi:10.1080/00036848700000004

Valdés B. Cigarette consumption in Spain: empirical evidence and implications for public health policy. Applied Economics 1993;25:149–56. Doi:10.1080/00036849300000019

Van Hurck MM, Nuyts PAW, Monshouwer K, et al. Impact of removing point-of-sale tobacco displays on smoking behaviour among adolescents in Europe: a quasi-experimental study. Tob Control 2019;28:401–8. Doi:10.1136/tobaccocontrol-2018-054271

Zelenka I. Tax policy impact on consumption of tobacco products in Croatia. Financial Theory and Practice 2009;:16.

Supplementary File 5: Quality of Included Studies

Cross Sectional Studies (n=35)
Average Score: 6/8

Author and year of publication	Score	Q1	Q2	Q3	Q4	Q5	Q6	Q7	Q8
**Agaku_2015**	5	Yes	Yes	No	N/A	Yes	Yes	No	Yes
**Alpert_2014**	6	Yes	N/A	Yes	N/A	Yes	Yes	Yes	Yes
**Asgeirsdottir_2014**	7	Yes	Yes	Yes	N/A	Yes	Yes	Yes	Yes
**Bogdanovica_2011a**	7	Yes	Yes	Yes	N/A	Yes	Yes	Yes	Yes
**Bruggink_2016**	7	Yes	Yes	Yes	N/A	Yes	Yes	Yes	Yes
**Chyderiotis_2019**	7	Yes	Yes	Yes	N/A	Yes	Yes	Yes	Yes
**Ciccarelli_2012**	4	Yes	N/A	Yes	N/A	No	No	Yes	Yes
**Costa_2006**	4	Yes	Yes	Unclear	N/A	No	No	Yes	Yes
**Del Bono_2018**	7	Yes	Yes	Yes	N/A	Yes	Yes	Yes	Yes
**Di Pietro_2018**	7	Yes	Yes	Yes	N/A	Yes	Yes	Yes	Yes
**Duffy_2006**	3	N/A	N/A	Yes	N/A	No	No	Yes	Yes
**Escario_2004**	5	Yes	Yes	Yes	N/A	No	No	Yes	Yes
**Feliu_2019**	7	Yes	Yes	Yes	N/A	Yes	Yes	Yes	Yes
**Filippidis_2014**	7	Yes	Yes	Yes	N/A	Yes	Yes	Yes	Yes
**Florkowski_1992**	6	Yes	N/A	Yes	N/A	Yes	Yes	Yes	Yes
**Gallus_2006**	7	Yes	Yes	Yes	N/A	Yes	Yes	Yes	Yes
**Hallingberg_2016**	7	Yes	Yes	Yes	N/A	Yes	Yes	Yes	Yes
**Hublet_2009**	7	Yes	Yes	Yes	N/A	Yes	Yes	Yes	Yes
**James_2015**	7	Yes	Yes	Yes	N/A	Yes	Yes	Yes	Yes
**Jimenez-Martin_1998**	5	Yes	Yes	Yes	N/A	No	No	Yes	Yes
**Jurges_2011**	7	Yes	Yes	Yes	N/A	Yes	Yes	Yes	Yes
**Kuipers_2017**	7	Yes	Yes	Yes	N/A	Yes	Yes	Yes	Yes
**Lidon-Moyano_2017**	5	Unclear	Yes	Yes	Yes	No	NA	Yes	Yes

**Martin Bassols_2016**	7	Yes	Yes	Yes	N/A	Yes	Yes	Yes	Yes
**Nociar_2016**	5	Yes	Yes	Yes	N/A	No	No	Yes	Yes
**Palali_2019**	4	Yes	N/A	Yes	N/A	No	No	Yes	Yes
**Pfortner_2016**	7	Yes	Yes	Yes	N/A	Yes	Yes	Yes	Yes
**Radfar_1985**	3	N/A	N/A	Yes	N/A	No	No	Yes	Yes
**Rathmann_2017**	7	Yes	Yes	Yes	N/A	Yes	Yes	Yes	Yes
**Ross_2014**	7	Yes	Yes	Yes	N/A	Yes	Yes	Yes	Yes
**Savage_2014**	4	Yes	N/A	Yes	N/A	No	No	Yes	Yes
**Schaap_2008**	7	Yes	Yes	Yes	N/A	Yes	Yes	Yes	Yes
**Schnohr_2008**	6	Yes	Yes	Yes	N/A	Yes	No	Yes	Yes
**Stavrinos_1987**	3	N/A	N/A	Yes	N/A	No	No	Yes	Yes
**Valdés_1993**	3	N/A	N/A	Yes	N/A	No	No	Yes	Yes

Critical Appraisal Checklist for Analytical Cross Sectional Studies (JBI Checklist)

Reviewer____Date__________________________________

Author____Year_________ Record Number_________

	Yes	No	Unclear	Not applicable
1. Were the criteria for inclusion in the sample clearly defined?	□	□	□	□
2. Were the study subjects and the setting described in detail?	□	□	□	□
3. Was the exposure measured in a valid and reliable way?	□	□	□	□
4. Were objective, standard criteria used for measurement of the condition?	□	□	□	□
5. Were confounding factors identified?	□	□	□	□
6. Were strategies to deal with confounding factors stated?	□	□	□	□
7. Were the outcomes measured in a valid and reliable way?	□	□	□	□

8. Was appropriate statistical analysis used? □ □ □ □

Overall appraisal: Include □ Exclude □ Seek further info □

Comments (Including reason for exclusion)

____

____

____

Time Series Studies (n=13)

Average Score: 9/11

Author and publication year	Score	Q1	Q2	Q3	Q4	Q5	Q6	Q7	Q8	Q9	Q10	Q11
**Hanewinkel_2008**	9	NA	Yes	Yes	Yes	Yes	Yes	Yes	Yes	Yes	NA	Yes
**Hyland_2009**	10	Yes	Yes	Yes	Yes	Yes	Yes	Yes	Yes	Yes	Unclear	Yes
**Lance_2004**	9	NA	Yes	Yes	Yes	Yes	Yes	Yes	Yes	Yes	NA	Yes
**Lemennicier_1998**	4	No	No	Unclear	Unclear	Yes	Yes	No	NA	Yes	No	Yes
**Ma_2018**	10	Yes	Yes	Yes	Yes	Yes	Yes	Yes	Yes	Yes	Unclear	Yes
**Marti_2014**	10	Yes	Yes	Yes	Yes	Yes	Yes	Yes	Yes	Yes	Unclear	Yes
**Nagelhout_2012**	10	Yes	Yes	Yes	Yes	Yes	Yes	Yes	Yes	Yes	No	Yes
**Ogloblin_2003**	10	Yes	Yes	Yes	Yes	Yes	Yes	Yes	Yes	Yes	No	Yes
**Ogloblin_2011**	10	Yes	Yes	Yes	Yes	Yes	Yes	Yes	Yes	Yes	No	Yes
**Olafsdottir_2015**	11	Yes	Yes	Yes	Yes	Yes	Yes	Yes	Yes	Yes	Yes	Yes
**Peng_2009**	6	NA	Yes	Unclear	Yes	Yes	Yes	Unclear	No	Yes	NA	Yes
**Raschke_2016**	10	Yes	Yes	Yes	Yes	Yes	Yes	Yes	Yes	Yes	Unclear	Yes
**Zelenka_2009**	9	NA	Yes	Yes	Yes	Yes	Yes	Yes	Yes	Yes	NA	Yes

Critical Appraisal Checklist for Time Series Studies (JBI checklist modified with Cochrane EPOC items)

Reviewer____ Date_________________________________

Author____ Year_________ Record Number_________

Yes No Unclear Not applicable

1. Were the criteria for inclusion in the sample clearly defined?	□	□	□		□
2. Were the study subjects and the setting described in detail?	□	□	□		□
3. Was the exposure measured in a valid and reliable way?	□	□	□		□
4. Were objective, standard criteria used for measurement of the condition?	□	□	□		□
5. **Was the intervention independent of other changes?**	□	□	□		□
**6. Was the shape of the intervention effect pre-specified?**	□	□	□		□
7. Were confounding factors identified?	□	□	□		□
8. Were strategies to deal with confounding factors stated?	□	□	□		□
9. Were the outcomes measured in a valid and reliable way?	□	□	□		□
**10. Was incomplete outcome data assessed adequately?**	□	□	□		□
11. Was appropriate statistical analysis used?	□	□	□		□

Overall appraisal: Include □ Exclude □ Seek further info □

Comments (Including reason for exclusion)

____

____

____

Longitudinal and Repeated Cross Sectional Studies (n=10)

Average Score: 7/8

Author and publication year	Score	Q1	Q2	Q3	Q4	Q5	Q6	Q7	Q8
**Asgeirsdottir_2016**	8	Yes	Yes	Yes	Yes	Yes	Yes	Yes	Yes
**Bogdanovica_2011b**	8	Yes	Yes	Yes	Yes	Yes	Yes	Yes	Yes

**Bruderl_2011**	5	Yes	Yes	Yes	Yes	No	NA	No	Yes
**Etile_2011**	8	Yes	Yes	Yes	Yes	Yes	Yes	Yes	Yes
**Forster_2001**	8	Yes	Yes	Yes	Yes	Yes	Yes	Yes	Yes
**Kaiser_2018**	8	Yes	Yes	Yes	Yes	Yes	Yes	Yes	Yes
**Leon-Gomez_2017**	7	Yes	Yes	Unclear	Yes	Yes	Yes	Yes	Yes
**Levy_2013**	4	No	No	Yes	Yes	No	NA	Yes	Yes
**Levy_2014**	4	Yes	No	Yes	Unclear	No	NA	Yes	Yes
**Nicolas_2002**	8	Yes	Yes	Yes	Yes	Yes	Yes	Yes	Yes

Critical Appraisal Checklist for Longitudinal and Repeated Cross Sectional Studies (JBI Checklist)

Reviewer____Date______________________________

Author_______________________________________Year_________ Record Number_________

	Yes	No	Unclear	Not applicable
1. Were the criteria for inclusion in the sample clearly defined?	□	□	□	□
2. Were the study subjects and the setting described in detail?	□	□	□	□
3. Was the exposure measured in a valid and reliable way?	□	□	□	□
4. Were objective, standard criteria used for measurement of the condition?	□	□	□	□
5. Were confounding factors identified?	□	□	□	□
6. Were strategies to deal with confounding factors stated?	□	□	□	□
7. Were the outcomes measured in a valid and reliable way?	□	□	□	□
8. Was appropriate statistical analysis used?	□	□	□	□

Overall appraisal: Include □ Exclude □ Seek further info □

Comments (Including reason for exclusion)

____
____
____

Cohort Studies (n=3)
Average Score: 9/11

Author and publication year	Score	Q1	Q2	Q3	Q4	Q5	Q6	Q7	Q8	Q9	Q10	Q11
**Serrano-Alarcón_2019**	9	Yes	Yes	Yes	Yes	Yes	Yes	Yes	Yes	Unclear	Unclear	Yes
**Jones_2015**	9	Yes	Yes	Yes	Yes	Yes	Yes	Yes	Yes	Unclear	Unclear	Yes
**Basu_2018**	10	No	Yes	Yes	Yes	Yes	Yes	Yes	Yes	Yes	Yes	Yes

Critical Appraisal Checklist for Cohort Studies (JBI Checklist)

Reviewer____Date_________________________________

Author____Year_________ Record Number_________

	Yes	No	Unclear	Not applicable
1. Were the two groups similar and recruited from the same population?	□	□	□	□
2. Were the exposures measured similarly to assign people to both exposed and unexposed groups?	□	□	□	□
3. Was the exposure measured in a valid and reliable way?	□	□	□	□
4. Were confounding factors identified?	□	□	□	□

5. Were strategies to deal with confounding factors stated? □ □ □ □
6. Were the groups/participants free of the outcome at the start of the study (or at the moment of exposure)? □ □ □ □
7. Were the outcomes measured in a valid and reliable way? □ □ □ □
8. Was the follow up time reported and sufficient to be long enough for outcomes to occur? □ □ □ □
9. Was follow up complete, and if not, were the reasons to loss to follow up described and explored? □ □ □ □
10. Were strategies to address incomplete follow up utilized? □ □ □ □
11. Was appropriate statistical analysis used? □ □ □ □

Overall appraisal: Include □ Exclude □ Seek further info □

Comments (Including reason for exclusion)

____

____

Quasi-Experimental Studies (n=1)

Average Score: 7/9

Author and publication year	Score	Q1	Q2	Q3	Q4	Q5	Q6	Q7	Q8	Q9
**Van Hurck_2019**	7	Yes	Yes	No	Yes	No	Yes	Yes	Yes	Yes

Critical Appraisal Checklist for Quasi-Experimental Studies (JBI Checklist)

Reviewer____Date________________________________

Author____Year_________ Record Number_________

	Yes	No	Unclear	Not applicable
1. Is it clear in the study what is the ‘cause’ and what is the ‘effect’ (i.e. there is no confusion about which variable comes first)?	□	□	□	□
2. Were the participants included in any comparisons similar?	□	□	□	□
3. Were the participants included in any comparisons receiving similar treatment/care, other than the exposure or intervention of interest?	□	□	□	□
4. Was there a control group?	□	□	□	□
5. Were there multiple measurements of the outcome both pre and post the intervention/exposure?	□	□	□	□
6. Was follow up complete and if not, were differences between groups in terms of their follow up adequately described and analyzed?	□	□	□	□
7. Were the outcomes of participants included in any comparisons measured in the same way?	□	□	□	□
8. Were outcomes measured in a reliable way?	□	□	□	□
9. Was appropriate statistical analysis used?	□	□	□	□

Overall appraisal: Include □ Exclude □ Seek further info □

Comments (Including reason for exclusion)

____

____

____

Supplementary File 6: Policy realms (Non-MPOWER measures) and descriptions

Policy realm	Description
1. Economic crises	Presence of economic crisis (e.g., downturn or recession) at the national or international level.
2. Education policy	Changes in national-level education indicators or policies related to access to education.
3. Macro-economic factors	Indicators of economic health at the national level; indicators of income equality or inequality.
4. Non-MPOWER tobacco regulations	Regulations or restrictions on tobacco consumption and manufacturing that are not captured by a specific WHO MPOWER measure.
5. Population welfare	Measures of population wellbeing and life satisfaction.
6. Public policy	Public policy programs that increase the general health and/or social security of individuals in a society, including mental health.
7. Sales to and by minors	Permissiveness or restriction of tobacco or cigarette sales to or by minors, including sales bans; reflects WHO FCTC Article 16.
8. Unemployment rate	Employment and/or unemployment rates at the national, regional, or youth-specific level.
9. Other	Includes:<br>- National-level tobacco production<br>- Population-level religiosity<br>- Country of residence in the former Western or Eastern Blocs

Supplementary File 7: Summary of key variables from included studies

Author and year of publication	Study period	Country/countries included	Study population group	Exposure	Outcome category	Effect on smoking rates
Agaku 2015	2012	Austria, Belgium, Bulgaria, Cyprus, Czech Republic, Denmark, Estonia, Finland, France, Germany, Greece, Hungary, Ireland, Italy, Latvia, Lithuania, Luxembourg, Malta, Netherlands, Poland, Portugal, Romania, Slovakia, Slovenia, Spain, Sweden, United Kingdom	General population	Health warnings on tobacco products	Other[1]	Decrease
Alpert 2014	2001-2011	Greece	Adults only	Cigarette excise tax increase	Cigarette sales	Decrease
Asgeirsdottir 2014	2007-2009	Iceland	Adults only	2008 Icelandic economic recession	Smoking prevalence	Decrease
Asgeirsdottir 2016	2007-2012	Iceland	Adults only	2008 Icelandic economic recession	Smoking prevalence	Decrease
Basu 2018	1958-2000	England, Wales	Adults only	Post-war education reforms	Smoking prevalence	Increase
Bogdanovica 2011a	2007-2008	Austria, Belgium, Bulgaria, Croatia, Cyprus, Czech Republic, Denmark, Estonia, Finland, France, Germany, Greece, Hungary, Ireland, Italy, Latvia, Lithuania,	General population	National Tobacco Control Scale score<br>National proportion of 18-24 year olds with low education<br>National unemployment rates<br>GDP per capita	Smoking prevalence<br>Smoking prevalence	Decrease<br>No effect<br><br>No effect<br>Decrease<br>No effect

[1] "Other" outcome category = Smoking-related behavioural response.

		Luxembourg, Malta, Netherlands, Poland, Portugal, Romania, Slovakia, Slovenia, Spain, Sweden		Income inequality<br>Proportion of population below 60% of national median income<br>National tobacco production quantity<br>Proportion of population reporting religion as important personal value<br>National average life satisfaction score<br>National gender equality score<br>National human development index score<br>National spending on social benefits<br>Perceived public sector corruption	Smoking prevalence<br>Smoking prevalence<br>Smoking prevalence<br>Smoking prevalence<br>Smoking prevalence<br>Smoking prevalence<br>Smoking prevalence<br>Smoking prevalence<br>Smoking prevalence<br>Smoking prevalence<br>Smoking prevalence	Increase<br><br>No effect<br>No effect<br><br>Decrease<br>Decrease<br>Decrease<br>Decrease<br>Decrease
Bogdanovica 2011b	2003-2009	Austria, Belgium, Bulgaria, Croatia, Cyprus, Czech Republic, Denmark, Estonia, Finland, France, Germany, Greece, Hungary, Ireland, Italy, Latvia, Lithuania, Luxembourg, Malta, Netherlands, Poland, Portugal,	General population	Cigarette affordability	Smoking prevalence	No effect

		Romania, Slovakia, Slovenia, Spain, Sweden				
Bruderl 2011	1984-2008	Germany	General population	Smoking bans<br>Smoking bans	Smoking intensity<br>Smoking prevalence	No effect<br>No effect
Bruggink 2016	2006-2013	Netherlands	Adults only	2008 Dutch economic crisis	Smoking prevalence	Decrease
Chyderiotis 2019	2007-2008	France	Adults only	2007 French smoking ban<br>2007 French smoking ban<br>2007 French smoking ban<br>2007 French smoking ban	Other[2]<br>Smoking intensity<br>Smoking intensity<br>Smoking prevalence	No effect<br>No effect<br>No effect<br>Decrease
Ciccarelli 2012	1871-1913	Italy	General population	Cigarette prices<br>Tobacco prices<br>GDP<br>GDP	Cigarette sales<br>Cigarette sales<br>Cigarette sales<br>Cigarette sales	Decrease<br>Decrease<br>Decrease<br>Increase
Costa 2006	1995	Austria, Belgium, Denmark, Finland, France, Germany, Greece, Ireland, Italy, Luxembourg, Netherlands, Portugal, Spain, Sweden, United Kingdom	General population	Anti-advertisement regulations<br>Tobacco prices<br>Informational campaigns<br>Smoking regulation policies	Smoking prevalence<br>Smoking prevalence<br>Smoking prevalence<br>Smoking prevalence	No effect<br>No effect<br>Decrease<br>Decrease
Del Bono 2018	1999/2000 and 2004/2005	Italy	General population	Smoking ban in indoor public places	Smoking prevalence	No effect

[2] "Other" outcome category = Smoking behaviors at home.

Di Pietro 2018	2005-2012	Italy	Adults only	Regional unemployment rates<br>Regional unemployment rates<br>2008 Italian economic recession<br>2008 Italian economic recession	Smoking intensity<br>Smoking prevalence<br>Smoking intensity<br>Smoking prevalence	Decrease<br>Decrease<br>Decrease<br>Decrease
Duffy 2006	1963 - 1999	United Kingdom	General population	Tobacco prices	Cigarette sales	Decrease
Escario 2004	1983-1993	Austria, Belgium, Denmark, Finland, France, Greece, Ireland, Italy, Netherlands, Spain, Sweden, United Kingdom	General population	Tobacco tax	Cigarette sales	Decrease
Etile 2011	1992-2003	France	Adults only	Post-war education expansion	Smoking prevalence	Decrease
Feliu 2019	2006-2014	Austria, Belgium, Bulgaria, Croatia, Cyprus, Czechia, Denmark, Estonia, Finland, France, Germany, Greece, Hungary, Ireland, Italy, Latvia, Lithuania, Luxembourg, Malta, Netherlands, Poland, Portugal, Romania, Slovakia, Slovenia, Spain, Sweden	General population	National Tobacco Control Scale score<br>National Tobacco Control Scale score	Smoking prevalence<br>Smoking prevalence (cessation)	Decrease<br>Decrease
Filippidis 2014	2006-2011	Greece	Adults only	2008 Greek economic recession	Smoking prevalence	Decrease
Florkowski 1992	1959-1985	Poland	General population	Cigarette prices	Cigarette sales	Decrease

Forster 2001	1909-1984	England, Scotland, Wales	Adults only	Tobacco tax<br><br>Tobacco tax	Smoking prevalence (age of initiation)<br>Smoking prevalence (years of smoking)	Decrease<br><br>Decrease
Gallus 2006	circa 2000	Albania, Andorra, Armenia, Austria, Azerbaijan, Belarus, Belgium, Bosnia and Herzegovina, Bulgaria, Croatia, Cyprus, Czech Republic, Denmark, Estonia, Finland, Macedonia, France, Georgia, Germany, Greece, Hungary, Iceland, Ireland, Israel, Italy, Kazakhstan, Kyrgyzstan, Latvia, Lithuania, Luxembourg, Malta, Moldova, Monaco, Norway, Poland, Portugal, Romania, Russia, San Marino, Serbia and Montenegro, Slovakia, Slovenia, Spain, Sweden, Switzerland, Tajikistan, Netherlands, Turkey, Turkmenistan, Ukraine, United Kingdom, Uzbekistan	General population	Cigarette prices	Cigarette sales	Decrease
Hallingberg 2016	2013/2014	Wales	Youth only	Written school tobacco policies	Smoking prevalence	No effect

Hanewinkel 2008	1991-2006	Germany	General population	Cigarette prices	Cigarette sales	Decrease
Hublet 2009	2005-2006	Austria, Belgium, Bulgaria, Czech Republic, Denmark, Estonia, Finland, France, Germany, Greece, Hungary, Iceland, Ireland, Italy, Latvia, Lithuania, Luxembourg, Malta, Netherlands, Norway, Poland, Portugal, Romania, Slovakia, Slovenia, Spain, Sweden, Switzerland, and Great Britain	Youth only	Public bans<br>Ratio of cigarette prices to GDP/capita<br>Vending machines policy<br>GDP per capita	Smoking prevalence<br>Smoking prevalence<br>Smoking prevalence<br>Smoking prevalence	Decrease<br>Decrease<br>Decrease<br>Decrease
Hyland 2009	2006-2007	Scotland	Adults only	Smoke-free air laws	Smoking prevalence	No effect
James 2015	1991-2012	England	Adults only	Expansion of UK post-compulsory education	Smoking prevalence	No effect
Jimenez-Martin 1998	1985-94	Spain	General population	Tobacco prices	Cigarette sales	Decrease
Jones 2015	1991-2009	England, Scotland	Adults only	Public place smoking bans<br>Public place smoking bans	Smoking intensity<br>Smoking prevalence	No effect<br>No effect
Jurges 2011	1999-2003	Germany	Adults only	Post-war education expansion	Smoking prevalence	Decrease
Kaiser 2018	2004-2014	Germany	General population	Local unemployment rates<br>Local unemployment rates	Smoking intensity<br>Smoking prevalence	Decrease<br>Increase
Kuipers 2017	2007-2011	Cyprus, Czech Republic, Denmark, Estonia, France, Finland, Germany, Greece,	Youth only	Laws restricting cigarette sales to minors	Smoking prevalence	No effect

		Hungary, Ireland, Latvia, Lithuania, Malta, Poland, Portugal, Slovak Republic, Slovenia, Sweden, United Kingdom				
Lance 2004	1996-2000	Russia	General population	Cigarette prices<br>Cigarette prices	Smoking intensity<br>Smoking prevalence	No effect<br>Decrease
Lemennicier 1998	1970-1994	France	General population	Anti-advertisement regulations	Cigarette sales	No effect
Leon-Gomez 2017	1999-2011	Spain	General population	Smoke-free air laws<br><br>Smoke-free air laws<br>Smoke-free air laws	Smoking prevalence (ex-daily smokers)<br>Smoking intensity<br>Smoking prevalence (daily smokers)	No effect<br><br>Decrease<br>Decrease
Levy 2013	1998-2010	United Kingdom	General population	Anti-advertisement regulations<br>Cessation treatment policy<br>Smoke-free air laws<br>Cigarette prices<br>Overall tobacco control policies<br>Health warnings on tobacco products<br>Mass media campaigns	Smoking prevalence<br>Smoking prevalence<br>Smoking prevalence<br>Smoking prevalence<br>Smoking prevalence	Decrease<br>Decrease<br>Decrease<br>Decrease<br>Decrease<br>Decrease<br>Decrease

					Smoking prevalence<br>Smoking prevalence	
Levy 2014	2006-2009	Poland	General population	Cigarette prices	Smoking prevalence	Decrease
Lidon-Moyano 2017	2012	Austria, Belgium, Bulgaria, Cyprus, Czech Republic, Denmark, Estonia, Finland, France, Germany, Greece, Hungary, Ireland, Italy, Latvia, Lithuania, Luxembourg, Malta, Netherlands, Poland, Portugal, Romania, Slovakia, Slovenia, Spain, Sweden, United Kingdom	General population	Advertising bans<br>Cessation treatment access and availability<br>Public place bans<br>Cigarette prices<br>National Tobacco Control Scale score<br>Health warnings on tobacco products<br>Public information campaign spending	Smoking prevalence<br>Smoking prevalence<br>Smoking prevalence<br>Smoking prevalence<br>Smoking prevalence<br>Smoking prevalence<br>Smoking prevalence	No effect<br>Decrease<br>No effect<br>No effect<br>Decrease<br>No effect<br>No effect
Ma 2018	2009-2011	Ireland	Adults only	Ratification of free secondary school education	Smoking prevalence	Decrease
Marti 2014	1997-2007	Switzerland	General population	National and regional tobacco control expenditures<br>National and regional tobacco control expenditures	Smoking prevalence (initiation)<br>Smoking prevalence (cessation)	Decrease<br>Decrease
Martin Bassols 2016	2005-2011	Spain	General population	Regional unemployment rates	Smoking prevalence	Increase

Nagelhout 2012	2004-2007	Ireland, England, Netherlands	Adults only	Smoke-free workplace legislation	Smoking prevalence	Decrease
Nicolas 2002	1957-1997	Spain	Adults only	Tobacco prices<br><br>Tobacco prices	Smoking prevalence (initiation)<br>Smoking prevalence (cessation)	No effect<br><br>Decrease
Nociar 2016	1995-2011	Bulgaria, Croatia, Cyprus, Czech Republic, Denmark, Estonia, Faroe Islands, Finland, Greece, Hungary, Iceland, Ireland, Italy, Latvia, Lithuania, Malta, Norway, Poland, Portugal, Romania, Russia, Slovakia, Slovenia, Sweden, Ukraine, United Kingdom	General populatio n	Residence in former Eastern Bloc<br>Residence in former Western Europe	Smoking prevalence<br>Smoking prevalence	Increase<br>Decrease
Ogloblin 2003	1996-1999	Russia	Adults only	Cigarette excise taxes	Smoking prevalence	Decrease
Ogloblin 2011	2000, 2009	Russia	Adults only	Cigarette prices	Smoking intensity	Decrease
Olafsdottir 2015	2007-2009	Iceland	Adults only	2008 Icelandic economic recession<br>2008 Icelandic economic recession<br>2008 Icelandic economic recession - reduction in income<br>2008 Icelandic economic recession - reduction in income<br>2008 Icelandic economic recession - reduction in working hours	Smoking intensity<br>Smoking prevalence<br>Smoking intensity<br><br>Smoking prevalence	Increase<br>Increase<br>No effect<br><br>Decrease<br><br>No effect<br><br>No effect

				2008 Icelandic economic recession - reduction in working hours	Smoking intensity<br><br>Smoking prevalence	
Palali 2019	2014	Austria, Finland, France, Germany, Ireland, Italy, Netherlands, Portugal, Spain, Sweden, United Kingdom	General population	Bans on advertising promotion and sponsorship<br>Cessation treatment policy<br>Smoke-free air laws<br>Tobacco prices<br>Health warnings on tobacco products	Smoking prevalence<br><br>Smoking prevalence<br>Smoking prevalence<br>Smoking prevalence<br>Smoking prevalence	No effect<br><br>No effect<br>No effect<br>Decrease<br>No effect
Peng 2009	1997-2006	Ukraine	General population	Volume of advertising<br>Cigarette affordability<br>Cigarette excise taxes<br>Cigarette prices	Cigarette sales<br>Cigarette sales<br>Cigarette sales<br>Cigarette sales	Increase[3]<br>No effect<br>No effect<br>No effect
Pfortner 2016	2005-2006	Austria, Belgium, Bulgaria, Czech Republic, Denmark, Estonia, Finland, France, Germany, Greece, Hungary, Iceland, Ireland, Italy, Latvia, Lithuania, Luxembourg, Malta, Netherlands, Norway, Poland, Portugal, Romania, Slovakia, Slovenia, Spain, Sweden, Switzerland, United Kingdom	Youth only	Advertising bans<br>Treatment for tobacco dependence<br>Public bans<br>Cigarette prices<br>Health warnings<br>Ratio of government spending on tobacco control to GDP per capita	Smoking prevalence<br>Smoking prevalence<br>Smoking prevalence<br>Smoking prevalence<br>Smoking prevalence<br>Smoking prevalence	No effect<br>No effect<br>No effect<br>Decrease<br>No effect<br>Decrease

[3] In Figure 5, this finding was coded as a decrease, because the relationship between volume of advertising and changes in smoking rates is directionally aligned with the MPOWER measure's theory of change that enforcement of tobacco advertising bans results in a decrease in smoking rates.

					Smoking prevalence	
Radfar 1985	1957-1968	United Kingdom	General population	Volume of advertising<br>Cigarette prices	Cigarette sales<br>Cigarette sales	Increase[4]<br>Decrease
Raschke 2016	1999-2010	Germany	Adults only	German Kindergeld parental benefit<br>German Kindergeld parental benefit	Smoking intensity<br>Smoking prevalence	Increase<br>Increase
Rathmann 2017	2009-2010	Austria, Belgium, Czech Republic, Denmark, Estonia, Finland, France, Germany, Greece, Hungary, Ireland, Italy, Latvia, Lithuania, Netherlands, Norway, Poland, Portugal, Slovakia, Slovenia, Spain, Sweden, Switzerland, United Kingdom	Youth only	National Tobacco Control Scale score<br>Youth unemployment rates<br>GDP per capita	Smoking prevalence<br>Smoking prevalence<br>Smoking prevalence	Decrease<br>No effect<br>Decrease
Ross 2014	1990-2010	Poland, Russia, Ukraine	General population	Cigarette excise taxes	Smoking prevalence	Decrease
Savage 2014	2002-2007	Ireland	Adults only	Smoking ban in workplaces	Smoking prevalence	No effect
Schaap 2008	1994-2004	Belgium, Czech Republic, Denmark, England, Estonia, Finland, France, Germany, Hungary, Ireland, Italy, Latvia, Lithuania, Netherlands, Portugal, Slovakia, Spain, Sweden	Adults only	Advertising bans<br>Treatment for tobacco dependence<br>Public place smoking bans<br>Cigarette prices<br>National Tobacco Control Scale score	Smoking prevalence<br>Smoking prevalence<br>Smoking prevalence<br>Smoking prevalence<br>Smoking prevalence	Decrease<br>Decrease<br>No effect<br>Decrease<br>Decrease<br>No effect<br>No effect

[4] See Footnote 3.

				Anti-smoking advertising expenditure<br>Health warnings on tobacco products	Smoking prevalence<br>Smoking prevalence<br>Smoking prevalence	
Schnohr 2008	2001-2002	Austria, Belgium, Croatia, Czech Republic, Denmark, Estonia, Finland, France, Germany, Greece, Hungary, Ireland, Israel, Italy, Latvia, Lithuania, Netherlands, Norway, Poland, Portugal, Russia, Slovenia, Spain, Sweden, Switzerland, Ukraine, United Kingdom	Youth only	Cigarette prices<br>Gini coefficient (income inequality)<br>GNI per capita<br>National ban on smoking in schools (vs. voluntary ban)<br>Minimum purchase age of 16 years<br>Minimum purchase age of 18 years	Smoking prevalence<br>Smoking prevalence<br>Smoking prevalence<br>Smoking prevalence<br><br>Smoking prevalence<br>Smoking prevalence	No effect<br>Decrease<br>No effect<br>Decrease<br><br>Increase<br>No effect
Serrano-Alarcón 2019	2004-2013	Austria, Belgium, Denmark, France, Germany, Italy, Netherlands, Spain, Sweden, Switzerland	Adults only	Cigarette prices<br>National Tobacco Control Scale score<br>Non-price/non-smoke-free TCS policies<br>Smoke-free policies	Smoking prevalence<br>Smoking prevalence<br>Smoking prevalence<br>Smoking prevalence	Decrease<br>Decrease<br>No effect<br>Decrease
Stavrinos 1987	1961-1982	Greece	General populatio n	Advertising expenditure by cigarette producers<br>Cigarette prices<br>Information provision and health education	Cigarette sales<br><br>Cigarette sales<br>Cigarette sales	No effect<br><br>No effect<br>Decrease

Valdés 1993	1964-1988	Spain	General population	Intensity of advertising<br>Cigarette prices<br>National Real Decreto legislation	Cigarette sales<br>Cigarette sales<br>Cigarette sales	No effect<br>No effect<br>Decrease
Van Hurck 2019	2007-2015	Austria, Belgium, Bulgaria, Croatia, Czech Republic, Denmark, Estonia, Finland, Greece, Iceland, Ireland, Latvia, Lithuania, Malta, Netherlands, Norway, Poland, Portugal, Romania, Slovakia, Sweden, Ukraine, United Kingdom	Youth only	National Tobacco Control Scale score<br>Implementation of point-of-sale tobacco displays<br>Minimum purchase age	Smoking prevalence<br>Smoking prevalence<br><br>Smoking prevalence	No effect<br>Decrease<br><br>Increase
Zelenka 2009	1998-2007	Croatia	General population	Cigarette prices<br>Excise duty<br>National anti-smoking legislation	Cigarette sales<br>Cigarette sales<br>Cigarette sales	Decrease<br>Decrease<br>Decrease

Supplementary File 8: Extracted non-MPOWER population-level exposures by exposure type and associated change in tobacco consumption

Policy realm	Exposure	Number of exposures and direction of change in tobacco consumption			
		Increase	Decrease	No effect	Total
Economic crises	2008 Dutch economic crisis		1		1
	2008 Greek economic recession		1		1
	2008 Icelandic economic recession	2	2		4
	2008 Icelandic economic recession –– reduction in income		1	1	2
	2008 Icelandic economic recession – reduction in working hours			2	2
	2008 Italian economic recession		2		2
Education policy	Expansion of UK post-compulsory education			1	1
	Proportion of 18–24-year-olds with low education			1	1
	Post-war education expansion		2		2
	Post-war education reforms	1			1
	Free secondary school education		1		1
Macro-economic factors	GDP	1	1		2
	GDP per capita		3		3
	Gini coefficient		1		1
	GNI per capita			1	1
	Income inequality			1	1
	Proportion of population below 60% of national median income	1			1
Non-MPOWER tobacco regulations	National anti-smoking legislation		1		1
	National Real Decreto legislation		1		1
	Smoking regulation policies		1		1
Population welfare	Average life satisfaction score		1		1
	Gender equality score		1		1
	Human Development Index score		1		1
Public policy	German Kindergeld parental benefit	2			2
	National and regional tobacco control expenditures		2		2
	Spending on social benefits		1		1
	Perceived public sector corruption		1		1
	Ratio of government spending on tobacco control to GDP per capita		1		1
Sales to and by minors	Laws restricting cigarette sales to minors			1	1
	Minimum purchase age	1			1
	Minimum purchase age of 16 years	1			1
	Minimum purchase age of 18 years			1	1
	Vending machines policy		1		1
Unemployment rate	Local unemployment rates	1	1		2
	National unemployment rates			1	1

	Regional unemployment rates	3			3
	Youth unemployment rates			1	1
Other	National tobacco production quantity			1	1
	Proportion of population reporting religion as important personal value			1	1
	Residence in former Eastern Bloc	1			1
	Residence in former Western Europe		1		1
Total		***14***	***29***	***13***	***56***

Supplementary File 9: Extracted MPOWER exposures by type and associated change in tobacco consumption MPOWER measures

Measure	Exposure	Number of exposures and direction of change in tobacco consumption			
		Increase	Decrease	No effect	Total
Protect people from tobacco smoke	2007 French smoking ban		1	3	4
	Enforceable national ban on smoking in schools		1		1
	Public bans		1	1	2
	Public place bans			1	1
	Public place smoking bans			3	3
	Smoke-free air laws		3	3	6
	Smoke-free policies		1		1
	Smoke-free workplace legislation		1		1
	Smoking ban in indoor public places			1	1
	Smoking ban in workplaces			1	1
	Smoking bans			2	2
Offer help to quit tobacco use	Cessation treatment access and availability		1		1
	Cessation treatment policy		1	1	2
	Treatment for tobacco dependence		1	1	2
Warn about the dangers of tobacco	Anti-smoking advertising expenditure			1	1
	Health warnings			1	1
	Health warnings on tobacco products		2	3	5
	Implementation of point-of-sale tobacco displays		1		1
	Information provision and health education		1		1
	Informational campaigns		1		1
	Mass media campaigns		1		1
	Public information campaign spending			1	1
	Written school tobacco policies			1	1
Enforce bans on tobacco advertising, promotion and sponsorship	Advertising bans		1	2	3
	Advertising expenditure by cigarette producers			1	1
	Anti-advertisement regulations		1	2	3
	Bans on advertising promotion and sponsorship			1	1
	Intensity of advertising			1	1
	Volume of advertising		2		2
Raise taxes on tobacco	Cigarette affordability			2	2
	Cigarette excise tax increase		1		1
	Cigarette excise taxes		2	1	3
	Cigarette prices		13	6	19
	Excise duty		1		1
	Ratio of cigarette prices to GDP per capita		1		1

	Tobacco prices		5	2	7
	Tobacco tax		3		3
Tobacco Control Score	National Tobacco Control Scale score		7	1	8
	Overall tobacco control policies		1		1
	National Tobacco Control Scale score excluding price and smoking bans			1	1
Total		***0***	***55***	***44***	***99***

Supplementary File 10: Impacts of all tobacco-related exposures on smoking rates, by age category under study.

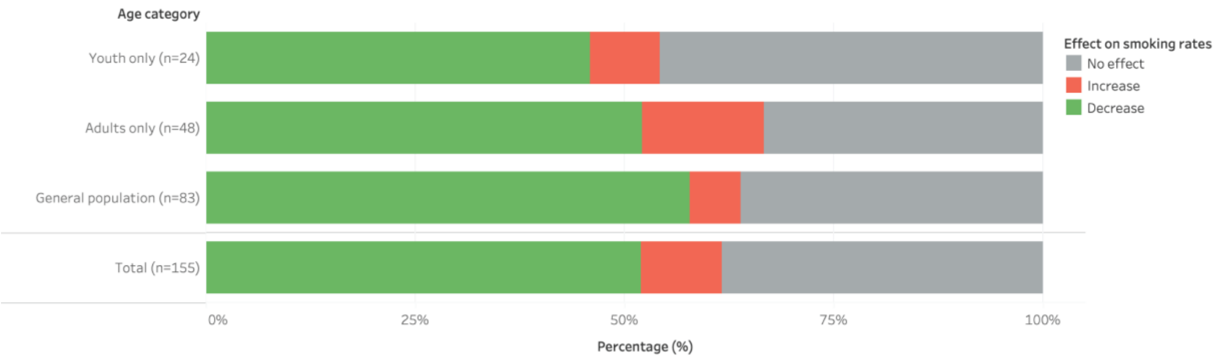

Supplementary File 11: MPOWER, Non-MPOWER, and Age Category Results After Removing 10 Lowest Quality Studies

MPOWER Measures

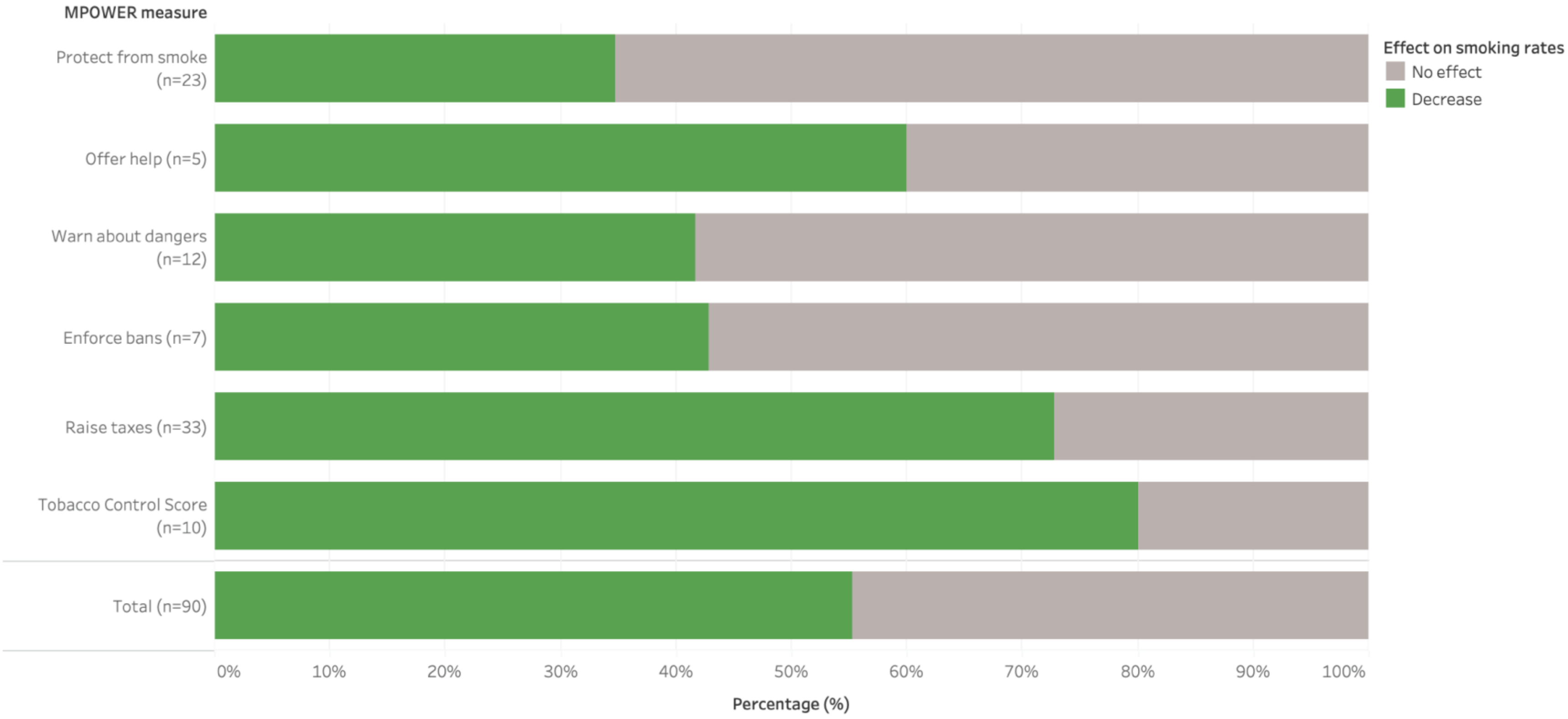

Non-MPOWER Policy Realms

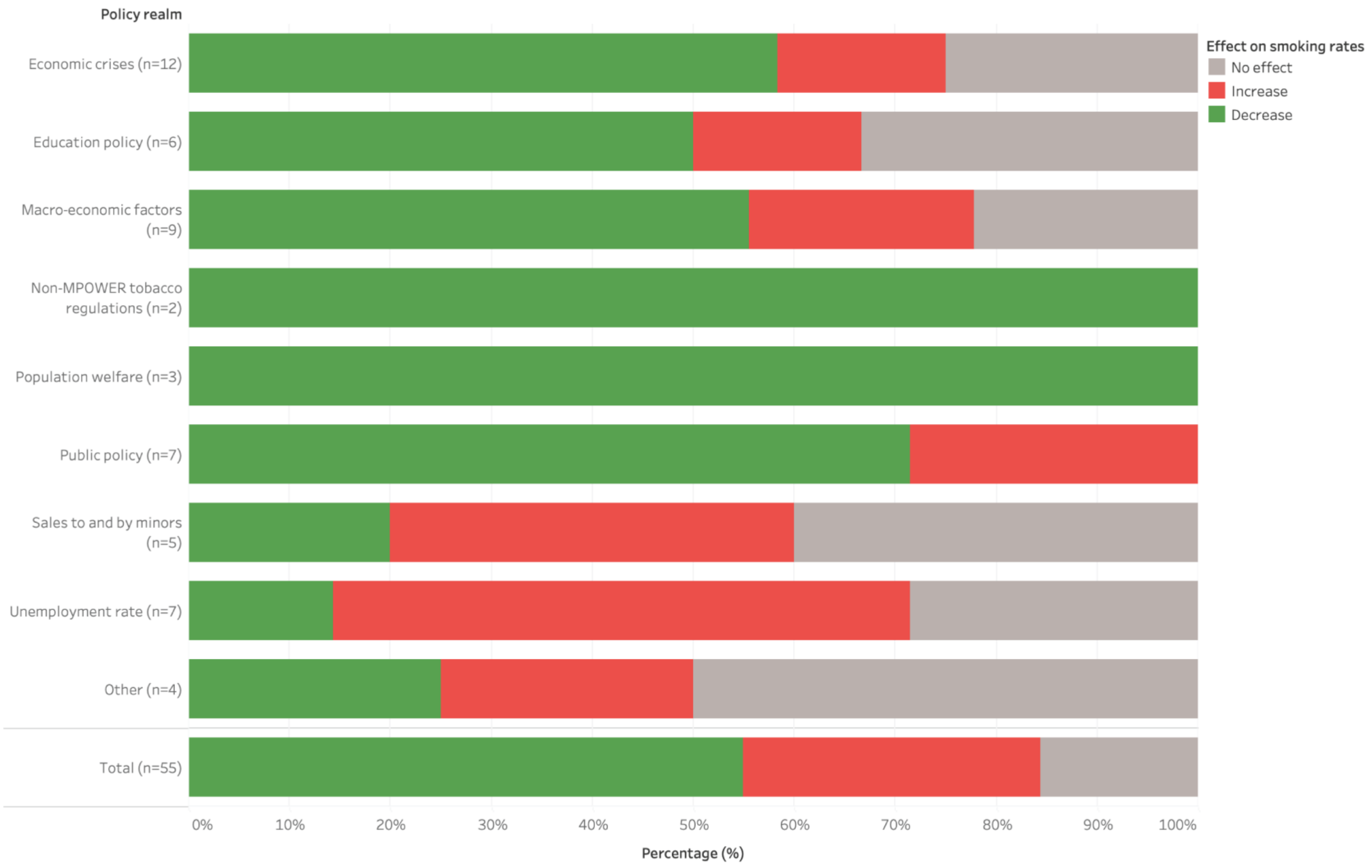

Age Category

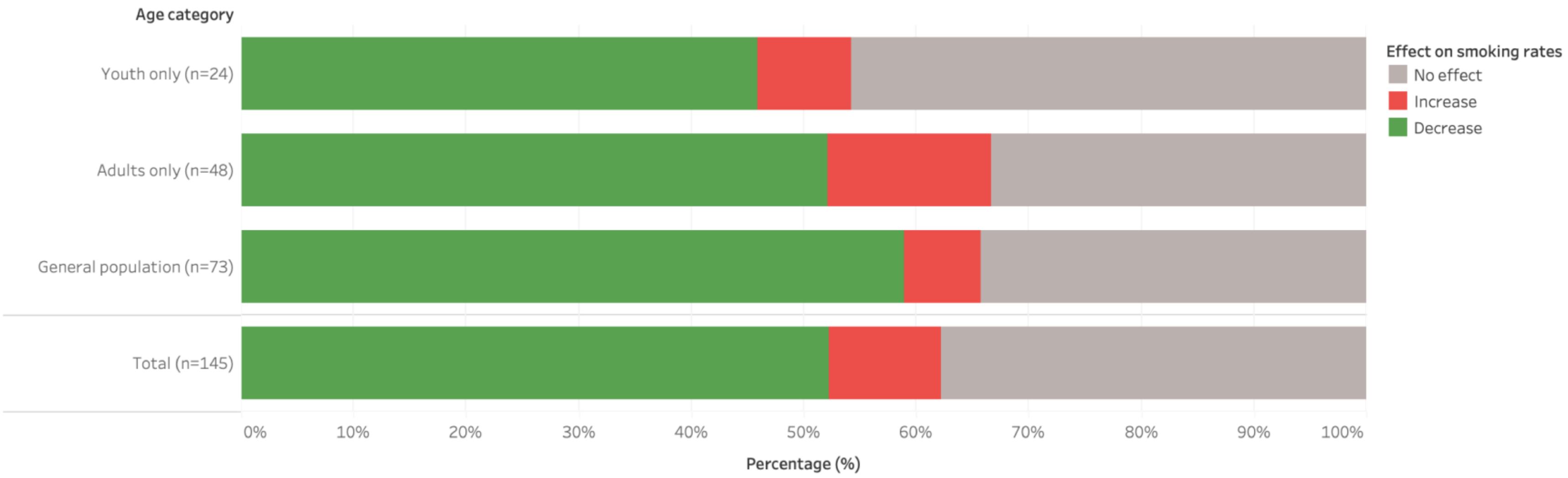

Supplementary File 11: PRISMA 2009 Checklist

Section/topic	#	Checklist item	Reported on page #
**TITLE**			
Title	1	Identify the report as a systematic review, meta-analysis, or both.	1
**ABSTRACT**			
Structured summary	2	Provide a structured summary including, as applicable: background; objectives; data sources; study eligibility criteria, participants, and interventions; study appraisal and synthesis methods; results; limitations; conclusions and implications of key findings; systematic review registration number.	2
**INTRODUCTION**			
Rationale	3	Describe the rationale for the review in the context of what is already known.	3
Objectives	4	Provide an explicit statement of questions being addressed with reference to participants, interventions, comparisons, outcomes, and study design (PICOS).	3
**METHODS**			
Protocol and registration	5	Indicate if a review protocol exists, if and where it can be accessed (e.g., Web address), and, if available, provide registration information including registration number.	NA
Eligibility criteria	6	Specify study characteristics (e.g., PICOS, length of follow-up) and report characteristics (e.g., years considered, language, publication status) used as criteria for eligibility, giving rationale.	5
Information sources	7	Describe all information sources (e.g., databases with dates of coverage, contact with study authors to identify additional studies) in the search and date last searched.	5
Search	8	Present full electronic search strategy for at least one database, including any limits used, such that it could be repeated.	Supplementary File 2
Study selection	9	State the process for selecting studies (i.e., screening, eligibility, included in systematic review, and, if applicable, included in the meta-analysis).	5
Data collection process	10	Describe method of data extraction from reports (e.g., piloted forms, independently, in duplicate) and any processes for obtaining and confirming data from investigators.	6

Data items	11	List and define all variables for which data were sought (e.g., PICOS, funding sources) and any assumptions and simplifications made.	Supplementary File 3
Risk of bias in individual studies	12	Describe methods used for assessing risk of bias of individual studies (including specification of whether this was done at the study or outcome level), and how this information is to be used in any data synthesis.	6
Summary measures	13	State the principal summary measures (e.g., risk ratio, difference in means).	NA
Synthesis of results	14	Describe the methods of handling data and combining results of studies, if done, including measures of consistency (e.g., I^2) for each meta-analysis.	NA

Section/topic	#	Checklist item	Reported on page #
Risk of bias across studies	15	Specify any assessment of risk of bias that may affect the cumulative evidence (e.g., publication bias, selective reporting within studies).	NA
Additional analyses	16	Describe methods of additional analyses (e.g., sensitivity or subgroup analyses, meta-regression), if done, indicating which were pre-specified.	NA
**RESULTS**			
Study selection	17	Give numbers of studies screened, assessed for eligibility, and included in the review, with reasons for exclusions at each stage, ideally with a flow diagram.	5
Study characteristics	18	For each study, present characteristics for which data were extracted (e.g., study size, PICOS, follow-up period) and provide the citations.	Supplementary File 7
Risk of bias within studies	19	Present data on risk of bias of each study and, if available, any outcome level assessment (see item 12).	8; Supplementary File 5
Results of individual studies	20	For all outcomes considered (benefits or harms), present, for each study: (a) simple summary data for each intervention group (b) effect estimates and confidence intervals, ideally with a forest plot.	7-10
Synthesis of results	21	Present results of each meta-analysis done, including confidence intervals and measures of consistency.	NA

Risk of bias across studies	22	Present results of any assessment of risk of bias across studies (see Item 15).	NA
Additional analysis	23	Give results of additional analyses, if done (e.g., sensitivity or subgroup analyses, meta-regression [see Item 16]).	NA
**DISCUSSION**			
Summary of evidence	24	Summarize the main findings including the strength of evidence for each main outcome; consider their relevance to key groups (e.g., healthcare providers, users, and policy makers).	11-12
Limitations	25	Discuss limitations at study and outcome level (e.g., risk of bias), and at review-level (e.g., incomplete retrieval of identified research, reporting bias).	12-13
Conclusions	26	Provide a general interpretation of the results in the context of other evidence, and implications for future research.	13-14
**FUNDING**			
Funding	27	Describe sources of funding for the systematic review and other support (e.g., supply of data); role of funders for the systematic review.	17

From: Moher D, Liberati A, Tetzlaff J, Altman DG, The PRISMA Group (2009). Preferred Reporting Items for Systematic Reviews and Meta-Analyses: The PRISMA Statement. PLoS Med 6(7): e1000097. doi:10.1371/journal.pmed1000097. For more information, visit: **www.prisma-statement.org**
